# Standardizing a unique renewable energy supply chain: the SURESC Model

**DOI:** 10.12688/f1000research.27345.1

**Published:** 2020-12-03

**Authors:** Emiliano Finocchi

**Affiliations:** 1 Temple University, Rome, Italy

**Keywords:** Research and Development, Carbon-free Technology, Energy Policy, Energy Shift, Renewable Energy, Energy Standards, Learning Curve

## Abstract

This study intends to dig into the renewable energy industry and drawing from research on learning curves and energy polices, proposes a way to speed-up the energy shift from our fossil-fuel dependency to a green economy. Even though standard economic frameworks suggest that markets and not policy makers should decide winners and losers, we urge to accelerate renewable energy competitiveness, proposing that by limiting the number of renewable technologies where resources are allocated to at government level, we reduce the time within which renewables will achieve technological price parity with fossil fuels. In turn, by analyzing the energy demand and supply curves, the study suggests that this action will also mediate the relation between quantity and price, shifting only the supply curve, leaving the demand curve unaffected. It continues by proposing the *standardization of a unique renewable energy supply chain* model, later defined as the SURESC model. For such, a deep analysis on existing green technologies will be performed proposing the implementation of a hydrogen through ammonia economy via ammonia for power as key factor for success. This is a preliminary study, first of its kind, intended to provide a holistic approach to a known problem.

## Introduction

### Geopolitics of the black gold

“Geopolitics is the battle for space and power played out in a geographical setting” [ 1, p.1].

**Figure 1.**
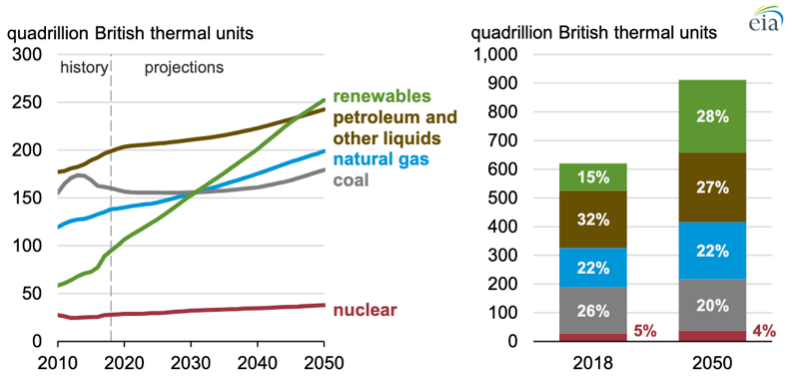
Global Primary Energy Consumption by Energy Source. (Source: U.S. Energy Information Administration, 2019.)

In the world’s geopolitical puzzle, energy plays a key role defining each piece and a driver to seek for global prosperity and security ^2^. Not all countries produce enough energy to impact the world's geopolitical scenario, but the global energy industry affects the geopolitical interests of all countries ^3^. In fact, for every 10 percent of oil price reduction, the world’s GDP grows 0.2 percent ^4^. As seen in Figure 1 ^5^, fuel such as petroleum and coal (fossil-fuels), account for more than half of the entire energy consumption source today, making it a strategic commodity for geopolitical bargains.

Forecasts for the near future do not seem to foresee any change, as even though renewable energy will largely increase by 150% by 2050, energy consumption will also increase by 50% driven by the Asian market ^5^, thus by 2050 petroleum and coal will still be accounted for 47% of the entire energy consumption source ( Figure 1). We need to change this trend!

By looking at Figure 1 we can understand the size of the economic interests for fossil-fuels behind all major economies and politics, which provide a great incentive to the world’s major conflicts (not necessarily wars) that are today spiked among the world’s biggest oil producers’ countries such as Iraq, Iran, Venezuela, Angola, Nigeria, Sudan, Saudi Arabia among others, ^1, 6^. Is no coincidence too that the two major oil producers in the world, US and Russia, have a significant role in these conflicts, both as mitigators or instigators, but always with a strong presence (for more information regarding this matter, consult literature on “The Dutch Disease”). Nigeria gave a name to this phenomenon: “the curse of the black gold” ^7^.

Not least, the impact to the environment for such amounts of fossil-fuel consumptions is well documented ^8– 11^ and accounts for today’s climate change ^11^. In fact, Höök and Tang ^11^ suggested that as fossil energy and climate change are strongly correlated, a solution must be found by treating them as “interwoven challenges necessitating a holistic solution” (p.1). Considering Höök and Tang ^11^ work, this study intends to develop a holistic approach towards this well-known phenomenon, and underlines the importance of a common, standardized eco-friendly solution where all major resources should be canalized in order to obtain a faster energy shift (from fossil to renewable) than forecasted nowadays. As we will discuss, no solution is perfect, but we need to balance pros and cons prioritizing global warming mitigation processes, which is one of our major responsibility as a developed society.

### And green we go…

“Renewable energy, often referred to as clean energy, comes from natural sources or processes that are constantly replenished” [ 12, p.2].

Climate change is attributed to high levels of carbon dioxide (CO _2_) emissions into the atmosphere as a result of the combustion of fossil-fuels for energy purposes ^8– 11^ that unbalance the CO _2_ cycle. Renewables don’t interfere with any natural cycles as are constantly replenished ^12^ (with exception of hydropower), providing Earth to maintain a natural balance. The most common and efficient renewable energies today are solar photovoltaic (PV), concentrated solar power (CSP), wind (onshore and offshore), oceanic (tidal and current), hydroelectric, biomass, and geothermal ^13^. Biomass for example, absorb CO2 from the atmosphere through a natural process, and release it under combustion, keeping the CO _2_ cycle balanced. Each one present advantages and disadvantages in energy potentials, but solar energy is by far the most abundant renewable energy source on earth ^14, 15^. Fact: every hour, the amount of power arriving from the sun to the surface of the earth, could provide for the global energy needs for one year ^16, 17^. 

Furthermore, with exception of geothermal and tidal energy, all other renewable energies are a transformation of solar energy into mechanical (hydroelectric), kinetic (wind and oceanic) and chemical (biomass) energies ^15^, and for each transformation, an efficiency factor has to be accounted for. A direct usage of the primary source could potentially mean a more efficient way to gather energy, but it depends on the technology used. Later, we will discuss on merits and demerits of each renewable energy source, focusing our work finding evidence to support that solar energy alone can potentially meet the world’s energy demand ^18^. Nevertheless, renewable energies present many technical issues, such as intermittence, geographical availability or engineering hazards, and therefore require green-energy storing facilities and green-energy dispensers. For example, solar energy depends on atmospheric events such as rain and cloudy days, besides earth rotation ^15^ and the energy produced must be stored for stabilization and distribution purposes.

### Dangers of unbalancing the energy geopolitics

“Even with renewable energy and energy efficiency, markets and not goodwill to arrest climate change, largely determine the path of investment” ^3^.

Even though renewable energy technologies are an extremely attractive energy replacement, as we will determine later in this research, fossil-fuels are hard to die. Business leads the way, and we will require a degree of human resources management similar to the one used in the cold war when this shift will happen ^3^. The randomization nature of the world’s natural resources distribution causes great inequalities and attract states and private organizations to aspire access grants to resources in foreign territories ^19^. In fact, even though many treaties and incentives on renewable energies have been signed widely (e.g. Kyoto protocol), countries don’t seem to reduce their carbon footprint. The US alone, for example, has passed from being the 3 ^rd^ biggest oil producer in 2008, to be the biggest oil producer in the world in 2019 ^20^, nearly doubling its production in 10 years.

### The need for a standardized energy stream model

“The disrupting elements of rapid change can be mitigated by common goals and a clear roadmap where incumbents join new players in implementing a low-carbon global energy transformation roadmap” [ 13, p. 20].

In the past years we have seen new renewable technologies and new renewable concepts rising at an astonishing rate. Today, we are producing (or potentially produce) green energy from wind, solar PV, concentrated solar PV, hydropower, biomass, geothermal, oceanic (tidal and current), cellulosic ethanol, artificial photosynthesis ^21^ and more, and yet, we haven’t been able to break our fossil-fuel dependency. This extremely segmented sector, made of endless technological possibilities, each with standalone projects most of the times immerged in regional realities, lacks a holistic approach ^11^ to boost-up and turn into the leading energy feedstock of the future. Standardization could be an important part of this process but is dependent on the maturity of the technology. Quebec’s Normalization Bureau ^22^ define standards as a set of agreements among players of a given industry defining characteristics and rules tailormade for that industry using benchmarks from field’s collective knowledge. Standardization means to understand and approach a problem in an agreed way (as a voluntary action), based on field knowledge and studies, and can leverage an innovation journey that can lead to excellence ^23^. Many studies on standardization have shown how standards are important to enhance the development of the industry involved ^22^, from medicine ^24– 26^, to HR ^27^, to Oil&Gas ^28^, to IT ^29, 30^, all industries have standardized themselves with time. Moreover, standardization diffuses knowledge, increases predictability and reduces uncertainty and risks ^23^, key strategic factors for small and medium enterprises (SME). But is standardization per se sufficient to incentive a green economy?

The energy sector is extremely complex, data is sometimes not available ^31^ and economic models depend on geopolitics, R&D advancements, competitive alternatives, subsidies and/or taxes, externalities, industry’s supply and demand elasticity and other factors that rapidly change with time ^32^, thus, are hard to determine. Moreover, the energy sector is heavily politicized, where usually the fossil fuel supply chain is subsidized (from supplier’s governments) and taxed (from demander’s governments) whereas green energy is generally subsidized to help it achieve competitive traits ^33^. Policy makers are trying to incentivize a green economy, but we are far from a real shift, as most forecasts have been missed, and we continue to increment the global usage of carbon energy despite all efforts. In 2017, total subsidies to the renewable energy upstream touched 166B$ globally (between private and public) ^34^, with an increment of 7.09% production from the previous year ^35^, equivalent to 442 TWh of extra power generated, and forecasts aim at 192B$ subsidies for 2030 ^34^. In the energy context these numbers aren’t even close to sustain an energy shift, as in 2017 global energy demand grew 543 TWh from 2016 ^36^ driven by the Asian markets, yet renewables alone couldn’t cover this need, lacking 11 TWh of power generation that has been supplied by carbon fuels. As suggested by the US Energy Information Administration ^37^, by following this trend our carbon emissions will continue to rise rather than fall, and our carbon dependency will be maintained ^1, 3, 5^. Furthermore, standard economic framework suggests policymakers to apply policies seeking for price parity in order to achieve competitiveness among energy sources. Nevertheless, price parity between green and brown energy may not be enough if economic models don’t account for fossil fuel price response to renewables, as governments producers of fossil fuel may use their price buffer created from taxes and royalties as a response to push fossil fuel prices yet lower ^31^. This suggests that in order to shift to a green economy, renewable’s price target should aim lower than the current fossil fuel benchmark and its economic models should foresee a strong economic response from its competitors. Technocrats and economists have spent thousands of hours creating models that would incentivize renewable energy deployment, and despite all our knowledge and efforts, we are behind in our green workplan, and our planet seems to be worming up faster than predicted ^38^. Global warming puts our external models (social, environmental, economic, etc.) under a large dose of stress which will consequently unbalance these systems. In non-stressful situations, it would be correct to leave to markets and time the burden of a “natural selection” of technologies, but in today’s environmental pressure, we can’t afford to wait for a technological breakthrough anymore, governments should model current economies with current technologies and regulations to create non-convex economies with convenience equilibria through R&D and taxes/subsidies. We need to act by maximizing all resources and create a coordinate globalized network of protocols that will help to speed-up this shifting process. This concept is not new to the literature and can be partially found in the Carbon Leakage theory ^39^, which states that in the absence of a global coordinated climate change policy, industries relying on fossil fuels as their primary energy source may relocate their premises in countries with less restrictive policies.

“Extremis malis extrema remedia” (Latin saying). The literature seam to point towards a mix use of renewable energies solution ^11, 13– 15, 17, 40^, and even though standard economic framework suggests that markets and not policymakers and energy geeks (like myself) should decide winners and losers, I propose that there is a direct positive relation between the number of renewable technologies subsidized at the government level and the speed with which these technologies can achieve price parity with fossil fuels. In other words, the greater the number of renewable technologies our governments subsidize, the more we increment the time within which these technologies will take to reach price parity with fossil fuels. Diminishing the number of renewable technologies to subsidize will speed-up the renewable deployment and renewable price parity of the selected technology with fossil fuels.

## Limiting the number of technologies

“Governments have been very creative in imposing price control, what is needed is to show the economic costs of those actions and evaluate fewer damaging alternatives” [ 41, p. 675].

Learning curves play an important role to understand maturing industries such as renewables, as the sizes of the investment needed for a certain renewable technology to reach technological price parity (leaning investment) with fossil fuels may define the time to achieve this goal ^42^. In this context, research and development (R&D) can diminish learning-by-doing investments in different ways ^43^, pushing the learning curve down by curve-shifting or curve-following (for more references on curve-shifting and curve-following please consult ^42^), diminishing the learning investment. Even R&D innovation catch-up could act as a catalyzer but requires continuous efforts through the establishment and adequate support of competence-creating units ^44^.

**Figure 2.**
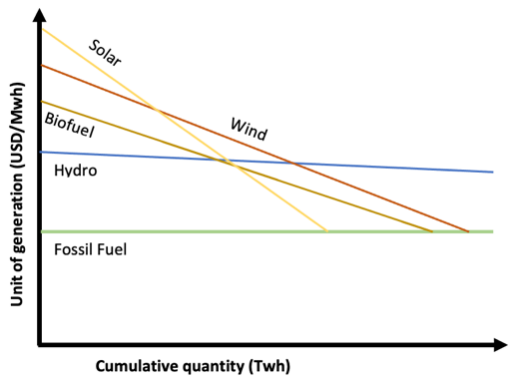
Green technologies learning curves based on learning-by-doing.

**Figure 3.**
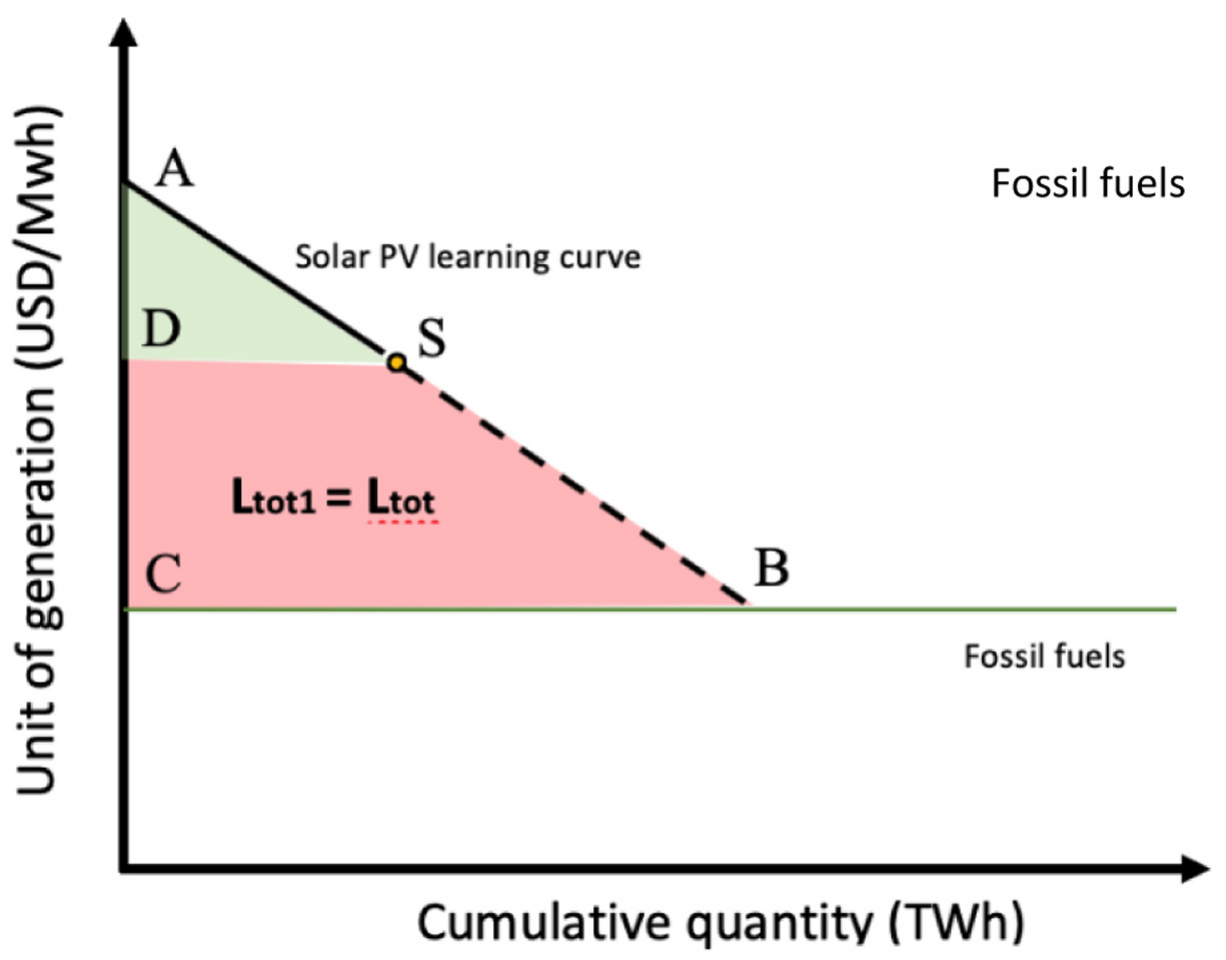
Solar PV learning curve based on learning-by-doing.

Let’s begin by analyzing Shayegh *et al.* ^42^ learning curve for Solar PV on a learning-by-doing bases (without R&D) represented in Figure 2. Fossil fuel unit cost of generation has been set at a fixed price of 50 USD/MWh, supposing it has reached it maximum learning point where cumulative quantity will not affect unit cost (benchmark). Solar PV has a learning quotient of 23%, intercepting fossil fuel line (price parity) with a future cumulative quantity (power deployed) at over 1000 TWh. On the Solar PV line, the continuous line represents historical data for Solar PV technology, the dot represents status quo, and the dotted line after the dot represent forecasts. The area between the Solar PV line (in all its length) and the fossil fuel line in Figure 3 ( $\bar{\mathrm{ABC}}$ ) is the total amount of learning investment needed to reach price parity. The area within the dotted line and the fossil fuel line represents what yet needs to be invested ( **Ltot1** = the difference between the total amount to be invested $\bar{\mathrm{ABC}}$ and the total amount invested so far $\bar{\mathrm{ASD}}$ ):

$$\bar{\mathrm{ABC}}-\bar{\mathrm{ASD}}=\bar{\mathrm{SBCD}}=Ltot1=\text{Ltot}$$

$\bar{\mathrm{ABC}}$ = Total learning investment to reach price parity

$\bar{\mathrm{ASD}}$ = ToFtal learning investment historically allocated

$\bar{\mathrm{SBCD}}$ **, Ltot1** = Total learning investment that needs allocation

**Ltot =** Sum of the total learning investments among selected technologies that needs allocation (only Solar PV, **Ltot = Ltot1**)

**Figure 4.**
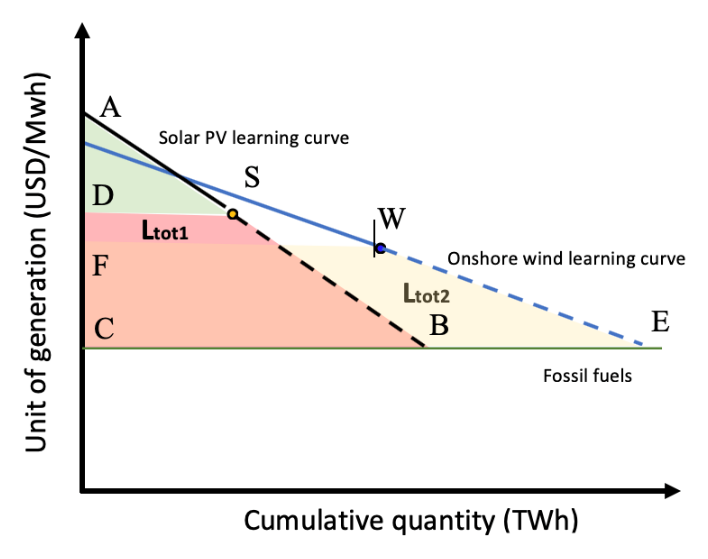
Solar PV and onshore wind learning curve based on learning-by-doing.

Now we introduce a second renewable technology, onshore wind ( Figure 4):

$$\bar{\mathrm{WECF}}=\text{Ltot2}$$

Ltot1+Ltot2=Ltot

By introducing **n** technologies, we have:

Ltot1+Ltot2+Ltot3+...+Ltotn=Ltot

Setting **Ltot1** as the smaller area, we have **Ltot2** = **Ltot1** + C1, where C1 is the difference between **Ltot2** and **Ltot1** ( **Ltot2** – **Ltot1**).

Therefore,

Ltot1+(Ltot1+C1)+(Ltot1+C2)+...+(Ltot1+Cn-1)=Ltot

$$\text{nLtot1}+\sum_{n-1}^{n-1}\mathrm{Cn}\;=\;\text{Ltot}$$

$\sum_{n-1}^{n-1}\mathrm{Cn}\;$ represent the sum of the area differences between the area $\bar{\mathrm{SBCD}}$ (solar PV learning investment needed) and the other renewable technologies areas, and therefore is a constant and can be simplified as **Ctot**. ***n*** represents the number of technologies.

nLtot1+Ctot=Ltot

*Ceteris paribus* but **n** and **Ltot**, so that **Ltot** becomes a function of **n**:


Ltot=Ltot1n+Ctot

Where **Ctot > 0**, **Ltot1 > 0** and **n > 0**.

This function represents the total amount of learning investment required for a set of renewable energy technologies to reach fossil fuels price parity. Usually, learning curves are indeed curves and not straight lines, but to the purpose of this exercise, the result would not change.

Continuing; every year both the private sector and the public sector allocate finite resources into the learning investment of renewable sources. These resources can be in the form of equity, subsidies, credit, loans, studies, etc. and are finite in nature (166B$ only in 2017’s subsidies as per Tylor ^34^). To simplify our calculations, let’s idealize a *constant* yearly resources allocation ( **RA**) to the learning investment **Inv**, and an equilibrium in the distribution of resources among technologies. This means that, every year ( **t**), the total learning investment **Ltot** required to achieve price parity decreases of a value of **RA**.

Ltot(t1)=Ltot(t0)-RA; Ltot(t2)=Ltot(t1)-RA;...;Ltot(tz*)=Ltot(tz*-1)-RA

$$\;*\text{z}=\frac{\mathrm{Ltot}}{RA}$$

**Figure 5.**
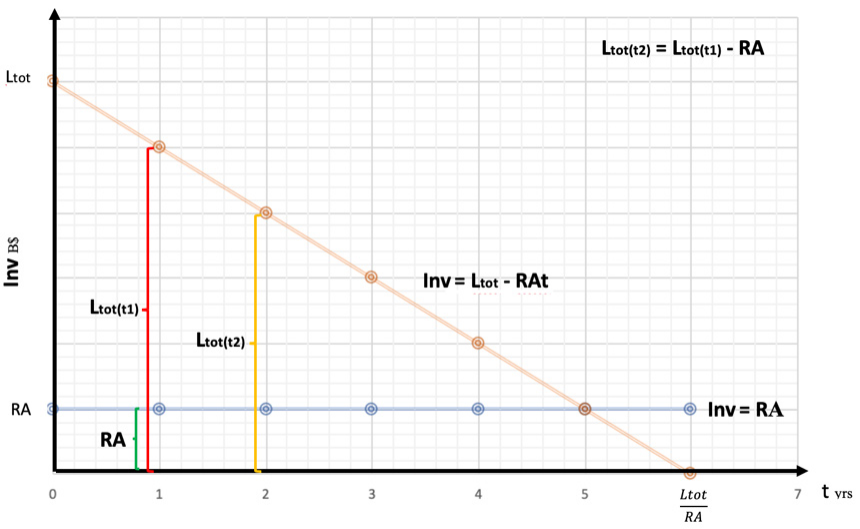
Effect of resources allocation into a single technology.

On the **Inv** axes of Figure 5, we have the amounts of resources (manhours, subsidies, quantity control protocols, etc.) converted into equivalent B$ as a function of time (t). The **RA** line represents the constant resources allocated year by year to the renewable industry by the private and public sector.

In Figure 5 we can see the amount of possible **RA** converted in USD as a function of time:


Inv=Ltot−RAt

We now substitute **Ltot**:


Inv=Ltot1n+Ctot−RAt

Price parity will be achieved when **Inv** = 0, this means when $\text{t}=\frac{\mathrm{Ltot}}{\mathrm{RA}},$ and by substituting **Ltot**:


$$\text{t}=\frac{\mathrm{Ltot}1n+\mathrm{Ctot}}{\mathrm{RA}}$$

As we can see, on a learning-by-doing process, as the number of technologies requiring learning investment increase, time to achieve price parity increases too. There is a direct, positive relation between time and the number of technologies where resources are allocated. Investing into R&D would shift the learning curve down, acting as a negative moderator between **t** and **n**. This means that as the sum invested into R&D increases, both time and learning investment decrease. Let’s demonstrate this claim:

Following Shayegh *et al.* ^42^ research, we find that investing in R&D will diminish initial investment costs **Ltot** and will push our learning curve down (for both curve-following and curve-shifting). In our model, it means that we diminish the initial **Ltot** by an **X** amount provided by the R&D process resulting into a new **R&DLtot** ^42^. Thus:


R&DLtot=Ltot−X

We now substitute **Ltot** with **R&DLtot:**


$$t=\frac{\mathrm{Ltot}1n+\mathrm{Ctot}-X}{\mathrm{RA}}$$

As **t** is a function of **n** (t(n)), **X** will act as a negative moderator to this function. To minimize **t**, we should allocate resources to R&D for **n=1**.

Taking into account that we are discussing at government level, another obvious result shown in this equation is that time is negatively related to the size of the resources allocated **RA**. This means that as more resources governments allocate, faster will parity price be accomplished (and vice versa).

We have found evidence to support that technological price parity between renewables and fossil fuels will be reached faster if limited government resources would be allocated to a restricted number of technologies. Reaching technological price parity means decreasing unit cost of generation price and increasing cumulative quantities too. We now need to apply this find to demand and supply renewable energy curves and see how the market would react by increasing and decreasing the number of technologies **n** we allocate resources to. Renewables produce electricity; thus, renewables’ commodity is electricity. General demand and supply linear curves in an *ideal market* are represented by the following inverted functions for both demand and supply:

- Demand: **P = a - bQd**
- Supply: **P = α + βQs**

*Ideal markets* require perfect competition, property rights well established, information availability, low externalities and no decreasing average costs as production increases ^32^. We will adapt from Dahl’s ^32^ coal demand and supply curves to electricity as main commodity, where in ideal markets the author describes **b** and **β** as the slope (elasticity), and **a** and **α** as the sum of the following parameters:

*Demand* **b**

- Price of substitutes to electricity (-Pse), such as natural gas used in stoves rather than electric stoves;
- Price of complements to the commodity (Pce), such as electric heaters vs gas heaters;
- Price for the technology for electricity use (+/-Tcu), such as electric cars vs diesel cars. This parameter may be positive or negative depending if the electric technology is cheaper than the alternative technology;
- Price of the output produced (-Pop). Prices of services using electricity as feedstock such as fun-parks, electric go-karts, and others;
- Energy policy (+/-Pol). It depends on the policies in place, positive for subsidies and negative for taxes;
- Number of buyers (-#buy)

*P=(Pse−Pce +/-Tcu+Pop+/-Pol+#buy)−b(Qd)

*Inverted curve: **Qd** is the demand quantity and **P** is the price

Parameters in the demand curve has no direct effect with the production technology. Demanders only know supplier’s selling price. The demand curve will not be altered by altering **n**.

*Supply* **β**

- Price of factors for producing electricity, such as labor and capital (-Pf);
- Price of similar goods that power plants could produce (-Psim). If oxygen or chlorine prices increase to a point where using electricity for electrolysis would provide more profit than selling electricity directly, green power plants could be enticed to change production;
- Price of by-products or complements of electricity production (Pb), which in renewables do not apply;
- Production technology (Tc), technical changes should reduce costs and increase production;
- government coal policies (+/-Pol), they could incentive or disincentive the industry;
- the number of sellers (#sell);

*P=(-Pf−Psim+Tc+Pb+/-Pol+#sell)+b(Qs)

*Inverted curve: **Qs** is the supply quantity and **P** is the price

**Tc** are the technical changes throughout time, representing technology advancement. As previously seen in learning curves, time to achieve technological price parity is directly related to the number of technologies **n** to allocate limited resources to. This means that by diminishing **n**, we increase technological advancement of maturing technologies and therefore we diminish the time between each technological change, resulting into an increase of **Tc**. In conclusion, **n** is negatively related to **Tc**, and Tc acts as a moderator to the **P(Qs)** function.

**Figure 6.**
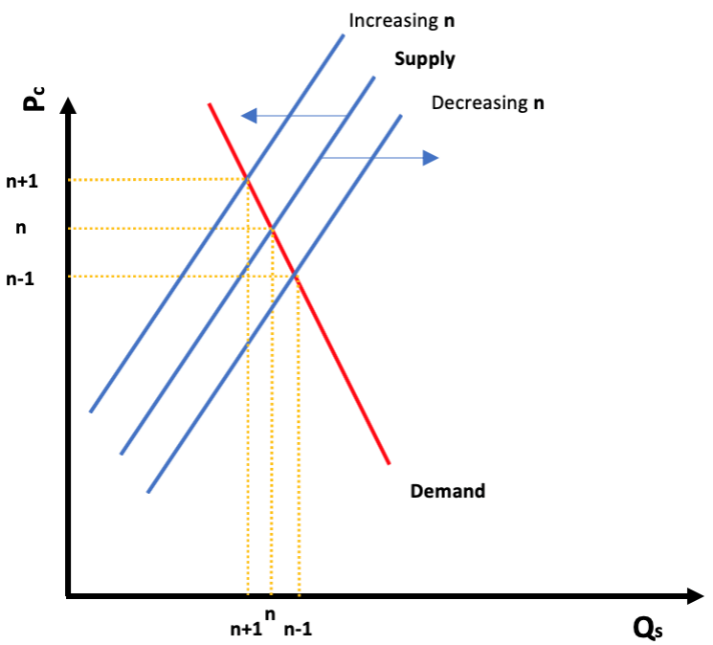
Ideal demand and supply renewable energy curves and shifting effect of selecting fewer technologies.

Figure 6 represents the shift in the supply curve when **n** varies. By increasing **n**, the supply curve will shift to the right, and vice versa, by diminishing **n**, the supply curve will shift to the left (in energy markets, both demand and supply are believed to be inelastic (b <1) ^32, 45, 46^). 

**Figure 7.**
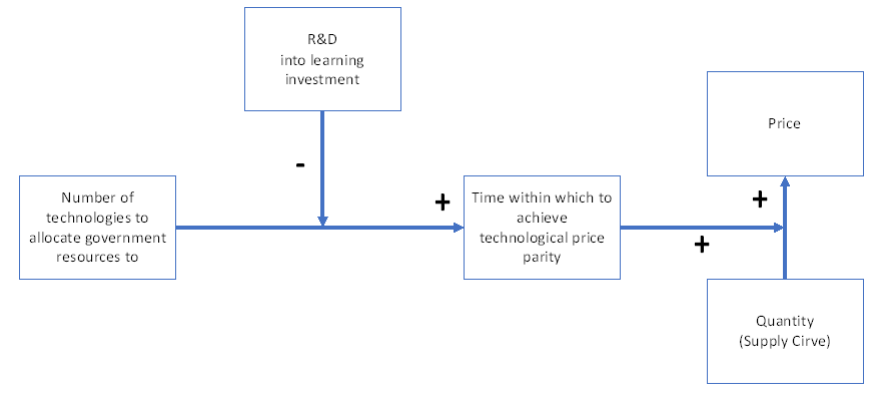
The SURESC Model.

We stated before that by allocating resources to fewer technologies we diminish the time within which renewables’ technological price parity with fossil fuels is achieved. Moreover, I found evidence to support that resource allocation to renewables’ R&D, will act as a negative moderator between the number of technologies receiving the resources and the time within which they will achieve this parity. Furthermore, adapting from Dahl ^32^ energy demand and supply curves, I found evidence to support that time to achieve technological price parity acts as a positive moderator in between the **P(Qs)** function. Figure 7 represents the final SURESC model.

It’s important to underline the fact that there is no perfect solution, that any technology will have its pros and cons, breakthroughs may come from excluded ones, and that economic frameworks strongly suggest a diversified portfolio of technologies. Nevertheless, a SURESC model will speed-up a green economy as it has become a priority due to a fast climate change. This research is a deep reflection to possible paths to follow by analyzing renewable energy streams and its technologies in order to build a first SURESC model.

### Energy streams

“Energy can neither be created nor destroyed in a system of constant mass, although it may be converted from one form to another” (first law of thermodynamics).

The physics of energy is fascinating. It can be manipulated endless of times at our own advantage, converted for specific needs such as transportation, and yet never vanish, it converts. The energy sector (non-renewable and renewable) are categorized into three main streams: upstream, midstream and downstream. EnergyHQ ^47^, supported by the Oklahoma Energy Resource Board, defines these streams as:

• *Upstream,* which refers to all the processes involving the exploration and production of energy. These include all engineering processes to find and reach the energy source like drilling, seismic, pumping, geological surveys, and all other processes that lead to the energy. In the renewable industry, upstream would be all those processes leading at the first energy transformation: solar to electric, wind to electric, geothermal to vapor, hydropower to electric, plantations to biofuel, oceanic to electric.

• *Midstream,* which refers to all the operations involving storage and transportation of the energy commodity to its energy transformation facilities (e.g. refineries, thermoelectric facilities, etc.), where is transformed into a usable source of energy intended for the final user (e.g. crude oil into gasoline or coal into electricity). Midstream includes pipelines, tankers, storage facilities, pumping stations and more. In the renewable industry, midstream includes storage and transportation of hydrogen generated by renewable systems, transportation of electricity through high tension lines, and the transportation of biofuel to end users.

• *Downstream,* which is the final sector of the energy streams, referring to all processes associated with the end usage of this commodity. In the oil sector, downstream would include all processes to produce diesel, gasoline, synthetic rubbers, and many more day-to-day products. In the renewable industry, downstream processes include hydrogen to electricity transformation and its distribution, electricity distribution into homes, industries and vehicles, and biofuels into usable fuels and its distribution.

**Figure 8.**
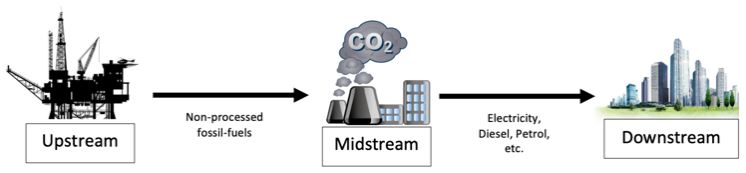
Graphical representation of the supply chain stream of fossil-fuel as feedstock.

There is the need to approach each stream (as shown in Figure 8) separately and understand what type of infrastructures and technologies exist, how these streams are connected to each other, and how energy is produced, transported and delivered into our homes, cars and industries. Energy is a very competitive and inelastic market, where companies have issues gaining market shares, thus, its supply chain must be demand driven and customer centric in order to survive ^25^. This study will have the same approach, a customer centric analyses (bottom-up), by starting to examine the energy flow from the clients’ needs (downstream) and follow the streamline in countercurrent (midstream) to the source (upstream), selecting a best-fit technology for the SURESC model form a client perspective.

## Discussion

### Downstream

“Transportation is believed to be currently responsible for nearly 60% of total world oil demand and will be the strongest growing energy demand sector in the future” [ 48, p.4587].

EIA ^37^ has divided the world energy consumptions into four main sectors: residential, commercial, transportation and industry. The industrial sector has the highest energy consumption, overall using over a third of the global energy production, followed by transportation, commercial and residential ^37^. Energy is delivered to end users in endless manners such as electricity, natural gas, steam, coal, diesel, gasoline, LPG, biofuel, and more. To make these deliveries possible, we have built a network of pipes, cables, trucks, facilities (substations, offices, maintenance centers, etc.) and services, that guarantee a constant flux in a vast geographical area full year-round. There are billions of dollars invested into these energy supply chain infrastructures, and most of them required invasive interventions to properties, land and the environment at a capillary level, and therefore must be taken into serious account before even ponder to replace them. Disrupting these systems would result in disrupting many economic interests, meaning that the financial damage of such operation could be an insurmountable barrier to achieve energy transformation, and needs to be carefully addressed. This is not a matter of goodwill, but a matter of available resources; markets probably can’t absorb such financial damage, especially in a relatively short period of time. Once again, economic resources are limited and should be used on a joint effort to create a competitive green economy, a global action for a global problem. The transportation sector is an exception to this complicated web, as its power system is intrinsic to the mobility nature of transportation, and there are small networks of infrastructures that sustain mobility that can be replaced with a smaller effort. All vehicles are built in factories and are fueled “on-the-run”, requiring a relatively simple network of energy dispensers accessible to mobile vehicles, while homes must have a constant and direct supply of energy, requiring a capillary delivery system that penetrate the entire territory. An energy shift implemented at the residential level would be much more invasive than a shift in the transportation sector, which, by the way, is already taking place, jump-started by Tesla’s success ^49^. We shall therefore not account for the transportation sector in this research as it will shape and adapt by itself according to the external environment and clients’ needs. For example, new car manufacturers such as Tesla are producing only electric cars, and conventional car manufacturers are betting on electric cars too (Toyota, Volvo, GM, Citroen, and others).

Finding a common energy denominator among final users would be a good starting point to identify a potential delivery system that could be integrated, rather than disrupted, into the SURESC model and minimize the number of technologies where resources are attributed to. We said that there are countless ways to deliver energy to factories, homes and offices, but without needing to analyze each and every one of them, we know that one is physically provided and needed to all, and powers most of our equipment and tools: electricity. Even though in some countries, electricity is not available to everyone (over 150 million Indians don’t have access to electricity ^50^), today it is widely used, its environmental impact is dependent exclusively to the production phase and can be converted into any other form of energy on demand (heat, kinetic, piezometric, etc.). Being able to provide clean, cheap and reliable electricity would be the solution to the disruptiveness problem which would require comparable small interventions to the existing systems and subsystems to replace other types of energies (such as natural gas and others), and therefore the impact of such would be minimal. Nevertheless, there are certain industries such as iron and steel, pulp and paper, basic chemicals and some non-metallic minerals ^51^ which require large amounts of direct heat in such a scale that existing electrical infrastructures wouldn’t be close to be able to supply their energy demands.

In conclusion, SURESC’s downstream solution would be to provide energy under electricity form to all end users by potentiating existing infrastructures and gradually abandon (or divert) other types of energy supply systems like natural gas, diesel or coal. SURESC’s downstream starts (and midstream ends) with the distribution system of electricity to end users, defining the electricity production phase as part of midstream. Next, we need to step-up the energy streamline into midstream, and analyze how the SURESC model could handle the transportation and storage of clean energy in its different form that will feed a clean power plant or energy dispenser. Once again, the use of non-disruptive technologies, or integrable technologies would be highly recommended.

### Midstream

“Electricity storage is considered a key technology to enable low-carbon power systems” [ 52, p. 81].

We have identified electricity as downstream energy for our SURESC model, but in midstream things can get a little confusing. In fossil-fueled systems, the production of electricity is a midstream definition, nevertheless, in renewable energy systems the production of electricity is considered to be an upstream, midstream and downstream matter according to the technology used. The difference resides on the primary source of energy exploited, which in fossil-fuels are hydrocarbons (all chemical energy) and sub-products (crude oil, natural gas, coal, etc.), which require extensive engineering processes to extract them from its natural deposits (upstream), later transported, stored and transformed into electricity (in midstream), while in renewables, the primary source varies from solar (PV), kinetic (wind), geothermal (thermic), chemical (biomass), among others, each one producing electricity in different stages. For example, PV, CSP and wind, produce electricity as first energy transformation (upstream), while biomass’ first energy transformation is into chemical energy (biofuel), later on transported, stored and transformed into electricity in midstream, while hydrogen-to-electricity, such as used in some vehicles, is transformed in downstream. This is not a mere definition problem, but a serious structural issue. Electricity has distance restrictions and can’t be transported too far from its source without substations injecting extra power into the grid (I will not dig further into the physics of electrical systems as is out of the scope of this research). This means that if we consider electricity production an upstream process in our SURESC model, it will require a decentralization of the upstream system, where production has to be built near consumption (which is a possibility), prioritizing power over energy, and midstream would comprehend electricity storage for stabilization purposes, with medium-low discharge duration and physical distribution from medium to small scale systems. Today, there are several tentative solutions in such direction, as seen with independent renewable projects in homes, offices and factories around the world, but unfortunately these systems supply less than 1% of the entire energy market. Meanwhile, considering electricity production a midstream process, would mean to collect and store energy at the source (wherever it may be) via an energy carrier, which would be shipped to powerplants around the world, prioritizing energy over power. This strategy would require a more centralized upstream system similar to the one seen in today’s fossil-fuel economy. These similarities are an incredible opportunity to adapt the existing infrastructures and accommodate the new energy carrier, with great financial savings and avoid disrupting many business sectors. If such option is available, existing infrastructures such as pipelines or energy dispensers among others, and logistics such as oil-tankers and gas-tankers among others, could be potentially upgraded to handle this new energy carrier. This process would yet be disruptive but less than a decentralized one, and an argument for further studies.

Ideally, considering a centralized model would largely benefit the finance of an eventual energy shift, but technically, we need to seek for energy parameters key to the centralization strategy and hope to find one that fits these parameters. For example, storing energy into lithium batteries is very efficient (up to 96%), cheap (compared to other technologies) and has no installation challenges. But energy and power separation is not possible with this technology, meaning that, in a centralized model, the entire battery should be transported to final destination, making it too costly and inefficient, and therefore not applicable, nevertheless a good fit for a decentralized one, where electricity can be stored and released on demand near points of consumption. Consequently, before deciding on matters such as centralization or decentralization, we need to analyze existing carbon-free energy storage technologies and energy carriers and compare them for different parameters that will provide us with enough elements to make such decision. For such, I will compare them to seven different parameters from three main areas: financial, technical and environmental. Financially, we need one parameter: the initial investment cost (CAPEX) which is sized based on power requirements to be released and is measured in $/kW. We will purposely dismiss the leverage cost of storage (LCOS) from financials as are too similar among each other ^52^ to be useful in our analyses. Technically, we need four parameters: roundtrip efficiency, lifetime cycles, transportability and installation challenges. Roundtrip efficiency measures how much energy is lost (money is lost) in a complete charging/discharging cycle measured in percentage. The lowest a roundtrip efficiency is, the lowest will be its financial feasibility. Lifetime cycles is the number of times the technology completes a charging/discharging cycle before reaching its physical end-of-life. Transportability is the ability to separate energy and power so that energy can be transported into a power facility elsewhere (transportation costs will be discussed later). Installation challenges are those technical challenges that require high degrees of engineering knowledge. Installation challenges is important parameter for accessibility purposes, as it may create an entry barrier not suitable for emerging economies and SMEs. Environmentally, we need two parameters: geographical availability, as not all environments have the right characteristics to host a technology, and land requirements, as some technologies may require large portion of land that may not be available. These seven parameters will provide us with enough elements to select our strategy (centralized or decentralized) and select our midstream technology for our SURESC model.

We have selected five main green storage technologies based on real-life models and applications:

• *Pumped hydropower storage (PHS)*: this mature system is very simple, PHS facilities store energy by elevating water form a lower level reservoir, to a higher-level deposit, gaining gravitational potential energy ^40^. When high demand energy peeks arise, the water is let to flow back to the lower reservoir which spins a turbine to produce the needed electricity ^53^. This system is usually used to balance demand peeks on a hydropower dam, but the very same principle could be used to store and manage energy demands for inconstant renewable energies. For example, it could use a portion of the electricity produced by solar panels during daytime, and release it during nighttime, when solar panels don’t produce. On a closed system, where all the energy is stored first, this process will also stabilize the output by adjusting the water flow downstream accordingly to the system’s short-term total production. Despite its high storage efficiency between 70% and 84% ^40^, pumped hydropower requires two major characteristics: large land availability with at least two significant elevation differences between plots, and water availability, which makes potential plant locations unequally distributed around the world ^40^. Moreover, there is a significant engineering work behind it to ensure the performance of the system accompanied by a large land-work activity. This is why this system is usually associated with hydroelectric dams and not built as independent systems. Due to its mature stage, implementing this technology is reasonably cost-effective, with an average CAPEX of 1.129 $/kW ^52^. In PHS energy is prioritized over power, making it ideal for seasonal storage or for medium to large power systems over 100MW ^40^. Transportability is not an option.

• *Advanced adiabatic compressed air energy store (AA-CAES)*: these systems have reservoirs in which air is pumped in and compressed to accumulate potential elastic energy ^40^, that can be returned at any point in time by using the compressed air to spin a turbine and generate electricity. One big challenge of this technology is due to the nature of its reservoirs, as the use of natural caves such as old salt deposit or gas depleted reservoirs can drastically diminish CAPEX and therefore make the investment feasible, where else, if a dedicated reservoir should be built, the CAPEX would spike and the investment could cease to make any economic sense ^40^. Thus, location is indeed a key factor for this technology. Another big issue is derived by the compression and decompression physics, meaning that each time a gas is compressed produces heat, and vice-versa, each time is decompressed cools down. This thermal “ups and downs” not only drastically diminish efficiency, as much of the energy is transformed into heat during loading, but this heat may disrupt the physical elements of the system. Standard CAES systems stabilize temperature releasing heat through a radiator while loading, and injecting heat through normal fossil fuel combustion (producing CO2) while unloading (diabatic process), and this is why this study will not consider standard CAES. In advanced adiabatic processes, the system stores heat in a heat storage facility during loading and release it while unloading ^40^. As heat can’t be stored for long periods, AA-CAES efficiency diminishes with time. Average CAES efficiency is 44% ^52^, but AA-CAES efficiency can reach 72% ^40^. Unfortunately, is hard to estimate installation costs for both CAES and AA-CAES, as only two systems are actually connected to the grid (Huntorf, Germany and McIntosh, Alabama), but if the reservoirs are of natural formation, an average of 871 $/kW can be estimated ^52^. In AA-CAES, as per PHS, energy is prioritized over power, but is limited to the size of the reservoir, making it a good choice for short seasonal storage or for medium to large power systems, between 10MW and 2500MW ^40^. Transportability is not an option.

• *Flywheel energy storage systems (FESS)*: these systems use energy to spin a mass (rotational kinetic energy), which is virtually frictionless due to its magnetic elevated bearing to avoid ground friction and placed in a vacuum to avoid air friction ^53^, and uses its moment of inertia in a later stage to invert the process and retrieve the energy stored [ 40, p. 58]. The total amount of energy that can be stored in this system is equal to the size of its rotatable mass by the speed in which it can spin; the heaviest and/or fastest, the more energy can store and vice-versa. There are two types of FESS, low-speed (under 10k revolutions per minute) and high-speed (up to 100k revolutions per minute), according to requirements ^54^. The system presents huge advantages: it can be built in factories and assembled in-loco anywhere needed, no special environmental requirements, carbon emission free, durable in time with low maintenance, long life cycles and an efficiency rate of up to 88% ^52^, nevertheless has two negative aspects: idle losses of up to 15% per hour (the system loses energy fast), and the need for strict security measures (a loose component can send tons of high-speed spinning mass out of control) ^40^. Its installation costs are in the lower range if compared to other energy storage technologies at 641 $/kW ^55^. In FESS, power is highly prioritized over energy, where the system needs to use its accumulated energy in short periods of time. It’s ideal for power stabilization purposes within hours for systems between 100 kW to 100 MW ^40^. Transportability is not an option.

• *Batteries*: it was 1800 when Alessandro Volta was accredited to have invented the first battery. A battery is a device capable of converting stored chemical energy into electrical energy ^53^. Anytime two chemical elements (or components) react together they exchange of electrons, and when these reactions happen in a controlled environment such as batteries, the system is able to direct these electrons to generate electrical power, and each of these reactions uniquely yield a performance, efficiency and safety characteristics ^40^. Based on the countless possible interactions between elements and components, with time we have developed many (or too many) battery systems. Even though batteries have become an essential component to all our electronics, from cell phones to cars, to baby toys, and our world would not be the same without these (portable and stationary) energy storing devices, we will not expand further on batteries’ technical description, as it would exceed the scope of this research. As stated, there are batteries of all “kind and shapes”, thus, to avoid distractions and based on previous studies ^53, 56– 58^, we have selected three main battery groups that have the potential to satisfy the energy storage requirements for this study: lithium-ion (Li-Ion) batteries, lead-acid batteries and vanadium redox-flow batteries.

◦ *The Li-Ion battery group*, per se a large group far from being homogenous, exchange Lithium ions (Li+) between the anode (made of “Lithium Metal Oxide”) and the cathode (usually made of graphite) ^56, 58^. The Li-Ion group comprise batteries such as Nickel-Manganese-Cobalt (NMC), lithium-Cobalt-Aluminum (NCA), Lithium-Iron Phosphate (LEP), Lithium Titanate (LTO) among others, each with different characteristics. Due to their mass-market usage, the production price of these batteries decreases on a monthly base, widening their implementation span. On average, these batteries have an incredible round-trip efficiency raging between 92% and 96% ^40^, shown to be the most efficient energy storing system seen so far in this study, and with an average CAPEX of 678 $/kW ^52^, makes this technology a front-runner for storing energy. Energy and power are balanced, but inseparable. This means that Li-Ion batteries are a good choice for very small, small and medium size power stations. Ideal for portable devices such as laptops or cars but are efficient with systems up to 100 MW too ^59^. Transportability is a valid option only in small devices.
◦ *Lead-acid batteries* are one of the oldest batteries on the market with over 150 years of existence. There are two types of these batteries: flooded (vented), which need a periodic water replacement, and valve-regulated (sealed) that use a valve to control inner pressure and prevent any electrolyte loss. Both have a very small lifecycle (sealed led-acid batteries a little longer) and are the heaviest batteries on the market. The electrolyte is made of aqueous sulfuric acid (H _2_SO _4_), the positive electrode is lead-oxide (PbO _2_) and the negative electrode is metallic lead (Pb) ^40^. Their roundtrip efficiency is high, but not as high as Li-Ion batteries, reaching 84%, and their implementation costs are about 625 $/kW ^52^. Like Li-Ion batteries, lead-acid batteries balance power and energy (inseparable) but are not so efficient with large systems. Due to its low price, is a good choice for small power stations up to only 25 MW ^40^. Transportability is a valid option for small systems like cars.
◦ *Vanadium redox-flow batteries (VRFB)* are a subsystem of the redox-flow batteries (RFB) family, which differ from normal batteries by having its electroactive materials dissolved in a liquid-state electrolyte. Although some designs also consist of having one of the electrolytes in a gaseous state (hydrogen/bromine cells), all reactions occur in ionic species in liquid solutions ^40^. The biggest advantage of these batteries consists in the separation of power and energy, where power can be scaled by scaling its cell stack design (by changing the electrode surface), and energy can be scaled by scaling the amounts of electrolyte stored in tanks ^40^. This provides the opportunity to develop batteries for large scale and long duration systems, which can be integrated into a power grid ^60^. Theoretically, energy could be transported by pumping around the electrolytes, where power can be stationary provided by pinpointing cell stacks according to the geographical needs. In this case the issue would be distance, as VRFB have a very low energy density between 0.081 MJ/Kg and 0.144 MJ/Kg, and therefore too expensive to transport. Transportability is possible but economically not feasible. Even though VRFB has some installation challenges, it also offers a simple heat control mechanism, as the system is fed by pumping the liquid electrolytes into the electrode stack, and in case of a system fault, switching off the pumps will stop all reactions. Moreover, even if more studies are required, the scientific community believes that this battery offers a potential environmentally friendly system, as none of its components present danger to the environment or a threat to humans ^61^. With an average initial investment cost of 829 $/kW ^52^, VRFB are ideal for small to medium size power stations up to about 100 MW ^40^.

Batteries can be built in factories and transported virtually anywhere with limited installation challenges. In VRFBs, energy could be transported separated from power, which can be controlled-released in a second moment. Nevertheless, batteries have some negative traits: they are heavy, can catch fire and their lifetime cycle is very short. For example, heat produced by normal batteries can be so intense that the battery itself can catch fire, as seen in some of the Tesla’s early models ^49^, and therefore are not suitable to transport large power systems. This is not so true for VRFB, as its lifecycle is high, its weight can be managed by managing its tanks and the heat be monitored by controlling the pumps. Worst, due to chemical degradation, batteries’ lifetime cycles are short if compared to any other energy storage technology. Again, this is not so true with Vanadium Redox-Flow batteries, which use liquid electrolytes that, in general terms, has lower chemical degradation.

• *Hydrogen*: this element has fascinated people for centuries and is considered to be the ultimate solution for energy and the environment ^62^, as with its combustion it releases only heat and pure water. The biggest advantage of this gaseous element consists in having the highest energy density values per mass of all energy carriers (142 MJ/Kg), more than triples crude oil and natural gas, and therefore is ideal for energy storage ^63, 64^. Nevertheless, on a volume scale the situation is reversed (8 MJ/Liter in its liquid form), where its values of energy per liter are much lower than of others ^65^. For example, in a confined space with equal environmental conditions, 1 kg of hydrogen has more than three times the energy of 1 kg of natural gas but occupies approximately 11m³ of space compared to approximately 1.1 m³ occupied by natural gas (depending on the composition of the natural gas). To make it more efficient, hydrogen needs to be compressed, but in equal conditions, natural gas will always have approximately 3.2 times more energy per volume than hydrogen and this is why hydrogen has never been a first choice for power generation, requiring large tanks and expensive logistics (even if 11 times lighter than air). Marketwise, hydrogen can yet be competitive as an energy carrier if the production price per volumetric energy density would achieve a cost of 3.2 times less than to exploit natural gas. Furthermore, hydrogen’s boiling temperature is -252,87 ˚C, requiring 45% of energy loss when transported in its liquid form ^64^. To worsen things, having the highest energy density among all energy carriers is its strength but also his curst, as is highly flammable and thus very dangerous to handle, requiring strong and costly security measures. But all is not lost… Studies suggest that by combining hydrogen with nitrogen present in the air, hydrogen can be stored inside the ammonia compound (NH _3_), a carbon-free substance that can potentially resolve many of hydrogen negative aspects ^55, 59, 66^. We will analyze ammonia as a sub product of hydrogen later. Hydrogen can be produced from fossil-fuels (grey hydrogen), from fossil-fuels combined with carbon capture (blue hydrogen) and directly from renewable sources (green hydrogen). Both grey and blue hydrogen produce high amounts of carbon dioxide as a byproduct, even though blue hydrogen captures it, yet storage is required ^64^. Unfortunately, 95% of all hydrogen produced today in the world is grey and blue hydrogen ^64^, but due to the scope of this research, we will focus on green hydrogen production only. Green hydrogen production process is simple and well documented ^63, 67^, obtained by water electrolysis (available virtually anywhere) and eventually re-electrified by combustion (releasing heat and H _2_O) or by using fuel cells. Unfortunately, its roundtrip efficiency is very low when compared to other storage technologies, reaching up to 42% with today’s technologies but with a theoretical potential to reach 54% ^53^. CAPEX to use this energy carrier is yet very high, calculated at 5,417 $/kw ^52^, thus having an insignificant market share in the energy industry. Nevertheless, the interest in hydrogen is growing by the day, and his potential role in the energy shift has been recognized by most governments ^64^, and efforts are made to bring CAPEX and LCOS prices down. Hydrogen is very versatile, but due to its low volumetric energy content, must be transported in large quantities, making it an acceptable choice for seasonal storage and large power stations over 50 MW ^59^. Some small applications in vehicles exist, but the market didn’t grow as expected.

◦ *Ammonia*: the base compound for fertilizers is ammonia, which its industrialized process uses fossil-fuels as feedstock for grey hydrogen production, contributing to approximately 1.3% global carbon emissions ^66^. Hydrogen production is key for these CO _2_ emissions, which we know can be produced by grey, blue and green processes. Despite this fact, ammonia production is a mature industry with an existing network of infrastructures, which is a great incentive for new implementations. In recent years, green ammonia has attracted international interests from many countries such as Japan, Australia, the US, Netherland and the UK ^59^, due to its ability to efficiently store hydrogen in its carbon-free formula (NH _3_) and be used as a possible energy carrier. Ammonia has a volumetric energy of 17.35 MJ/Liter, comparable to those of liquified natural gas (LNG), standing at 20.8 MJ/Liter, but with an incredible advantage: it can be liquified at atmospheric temperature through simple compression at 0.8 MPa (LNG requires temperatures of -160°C). The production process gets its name from its inventors, Fritz Haber and Carl Bosh (also known as Haber-Bosh process) and consists in reacting hydrogen with atmospheric nitrogen (accumulated through an air separator) using a metal catalyst under certain pressures and temperatures (we will not dig further into the Haber-Bosh process as will not provide a contribution to this research). The Haber-Bosh process is a carbon free process easy to implement. Security wise, ammonia is very toxic to humans and to the environment (graded 3 out of 4 of the hazardous scale form the US National Fire Protection Association) but has a low flammability with helps the handling. Nevertheless, it requires a constant and costly monitoring measures. Furthermore, other security challenges are derived from the reduction of NOx and unburned ammonia from non-performing power generators, both toxic to life, as NO _2_ is proven to aggravate cardiovascular and respiratory diseases, but as a consolidated industry, all protocols and safety measures are well tested and successful in both synthesis and combustion ^59^. In terms of its roundtrip efficiency, ammonia is more efficient if used directly to produce electricity (fired or through a fuel cell), avoiding dehydrogenization which requires an additional loss of 13% efficiency ^64^. This process can reach a roundtrip efficiency of up to 72% ^50^, nearly doubling pure hydrogen. In terms of CAPEX, ammonia storage is surprisingly low if compared to hydrogen, with an investment cost between 1,350 $/kW and 1,690 $/kW ^59^. Ammonia balances hydrogen best and worst qualities, providing a good volumetric energy content, and an easy handling logistics. Therefore, is a very good choice for seasonal storage and for medium to large power stations over 10 MW ^59^.

**Figure 9.**
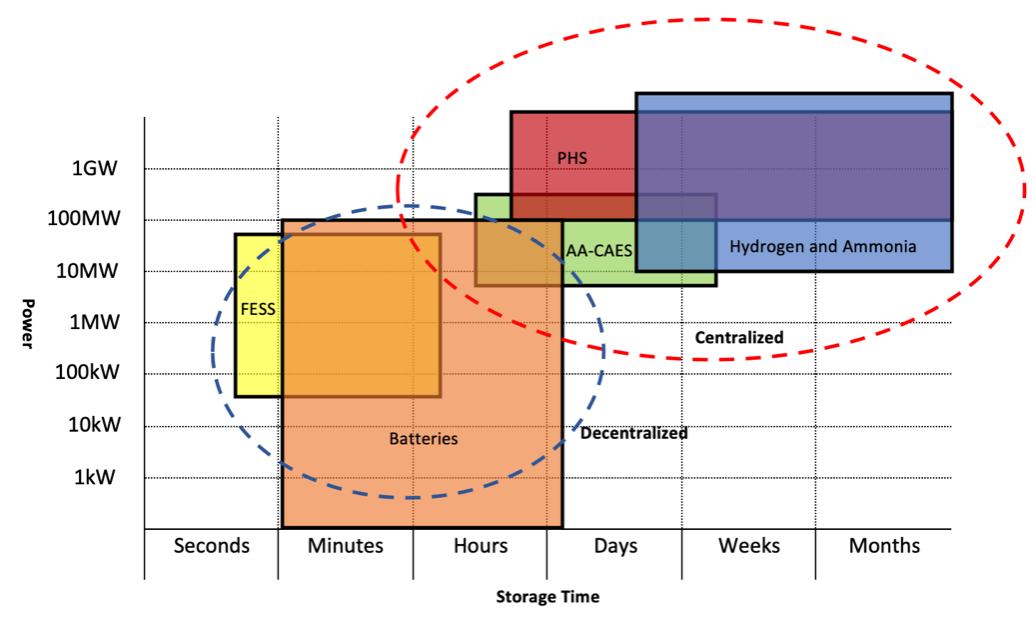
Energy vs power for renewable storing technology.

Based on the above characteristics, we can now make assumptions on which strategy is a better choice between centralized or decentralized (as graphical represented in Figure 9). A centralized model would imply prioritizing energy over power, where energy is transformed and stored in a remote location, transported to a power station, which than produces high power electricity for a large-scale distribution. Therefore, a centralized model would require energy over power prioritization and transportability. On a decentralized model, where power generation is brought close to the end user, power is prioritized over energy, as electricity is released and distributed in loco in smaller networks. Therefore, a decentralized model would require power over energy prioritization without the need for energy transportability.

As stated, transportability (energy and power separation) is another key decisional factor. Table 1 shows two potential energy storing technology for a centralized model, hydrogen and ammonia, and six for a decentralized one, where VRFB could have played a role in both scenarios if its energy density would have been higher. The financial aspect is also a very important part of our analyses, as if too high, all efforts to diminish a financial impact of using existing infrastructures may be vanished by a high CAPEX. Fortunately, this is not the case, as all technologies present a small variance among their CAPEX but hydrogen, which is much higher, whereas ammonia can provide a good alternative. Nevertheless, hydrogen CAPEX can notably diminish with the conversion of existing logistics and storing of natural gas facilities into hydrogen facilities ^68^. Many studies on this matter have concluded that this conversion is possible and not expensive ^69– 73^, as hydrogen behaves similar to natural gas, thus it can be handled in the same way with the right cautions. Again, ammonia would be preferred to hydrogen per se as its volumetric energy is greater and transportation much cheaper.

**Table 1.** Renewable energy sources storage technologies’ main traits.

	CAPEX $/kW	Round- trip Efficiency	Lifetime Cycles	Installation Challenges	Transp.	Geographical Availability	Land Requirements	
**PHS**	1,129	70% - 84%	33,250	Very High	No	Medium	Very High	**Decentralized**
**AA-CAES**	871	44% - 72%	16,250	Very High	No	Low	Low	
**FESS**	641	Up to 85%	143,402	Medium	No	Very High	Very Low	
**Batteries** **• Li-ion**	678	92% - 96%	3,250	Very Low	No	Very High	Very Low	
**• Lead-Acid**	675	Up to 84%	1,225	Very Low	No	Very High	Very Low	
**• VRFB**	829	Up to 85%	8,272	Medium	No	Very High	Very Low	
**Hydrogen**	5,417	Up to 42%	20,000	Medium	Yes	Very High	Low	**Centralized**
**• Ammonia**	1,350 – 1,690	Up to 72%	N.D.	Low	Yes	Very High	Low	
	**Financial**	**Technical**				**Environmental**		

Another concern is hydrogen’s low roundtrip efficiency at 42%, which with ammonia once again goes up to 72%. Ammonia requires only hydrogen and atmospheric air to be produced, making it available virtually anywhere the upstream source is most available. On the eventuality of a decentralized strategy, Li-Ion batteries and FESS have the best characteristics for power stabilization purposes, while PHS and AA-CAES are restricted by the environment but ideal for seasonal storage.

**Figure 10.**
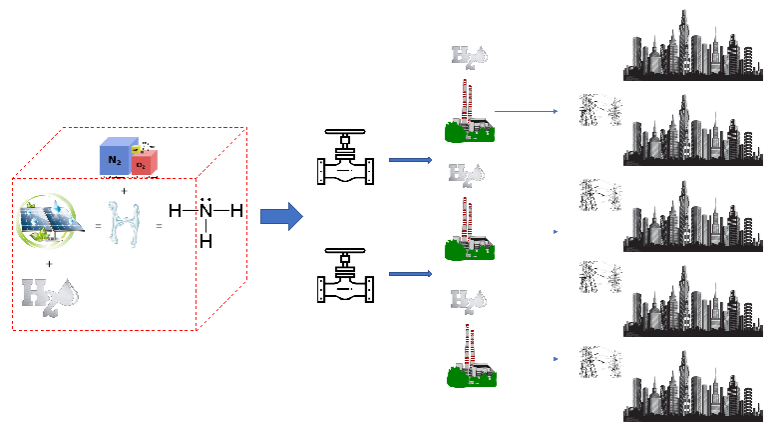
Graphical representation of a centralized model.

In conclusion, choosing a centralized strategy (as shown in Figure 10) over a decentralized one is preferred and possible, where hydrogen per se would not satisfy technical and financial parameter, hydrogen embedded into ammonia will, even if more detailed studies are required. “Hydrogen through ammonia economy via ammonia for power” [ 59, p. 96], this will be our SURESC’s midstream model. SURESC’s midstream starts (and upstream ends) with the storage of green ammonia, leaving its production to the upstream, followed by transportation to the centralized power station network. Next, we need to step-up once again the energy streamline into upstream and analyze how green ammonia can be produced efficiently and above all, carbon-free. Once again, the use of non-disruptive technologies, or integrable technologies would be highly recommended.

### Upstream

“The dissemination of renewables is related to the development of several individual technologies, besides energy policies and market opportunities. A lot of unused potential is associated with renewables throughout the world” ^74^.

We have agreed upon a centralized SURESC model that will use (as much as possible) the existing fossil-fueled energy supply chain infrastructures, and our energy carrier, ammonia, would have to be green. As stated, green ammonia is produced using green hydrogen from water electrolysis and nitrogen separated from the atmosphere, therefore requiring two main futures: water and electricity that will power both hydrogen production and the air separator for the nitrogen. As water and air are globally available will not be a matter of concern at this stage. In terms of electricity, its production shall be seen as a mere transit process within the hydrogen production phase, within ammonia production facilities, and it must be green. Selecting the right renewable energy for electricity production in upstream is again key to maximize efficiency and minimize costs to our overall SURESC model. This section will be comparing specific characteristics and parameters (financial, technical and environmental) of existing technologies, as we have done with storage technologies in midstream. For the financial parameters we have to consider CAPEX ($/kW) and the levelized cost of electricity (LCOE, measured in $/kWh). LCOE is a similar concept as LCOS, which is the breakeven cost of operation to produce electricity, often referred to as convenient summery measure to compare generating technologies ^5^. Technically, we need to know how efficient each technology is in converting green energy into electricity, and how complicated would be to achieve this goal (installation challenges). On the environmental side, we need to know the geographical availability, as it will impact logistics, and land requirements, which is today a global environmental issue and may spike CAPEX.

Today, proven renewable technologies are the following:

• *Solar, or photovoltaic (PV):* this technology uses semiconductors cells (such as silicon) to transform photons (sunlight) directly into electricity, from it the name “photo” meaning light and “voltaic” meaning electricity ^75^. The principle behind is simple, once solar light strikes a PV cell, a portion of its energy is transferred to the semiconductor, which knocks electrons loose ^75^. These cells have a magnetic field which then drive these loose electrons in a flow, producing a current measurable in wattages. Even though sunlight is by far the most abundant energy source on the planet ^14, 15^, photovoltaic technology has a low efficiency rate that stands between 15% ^18^ and 30% ^76^. Nevertheless, San Diego University Professor Murphy ^18^ believes that even 15% efficiency is enough for our needs, and has calculated that potentially, up to 44% of sunlight energy arriving to the ground can be recoverable (transformed into electricity) with PV cells. This means that PV cells can potentially increase their efficiency with new technological advancements. Another issue that may arise with PV is that the amount of electricity generated by surface area is low, meaning that the amount of land needed to generate high wattages of electricity is also high, creating a visible impact to the environment. Once more, due to its source availability, these panels can be installed in remote areas with low environmental impact such as deserts, where the strength of sunlight is higher. Another extremely important factor is the price. Based on the 2018 International Renewable Energy Agency report, PV has decreased its cost on average 13% on annual basis, and possibly by the end of 2020 the average cost of electricity produced with PV cells will be compared to the least-cost fossil fuel alternative. The cost per kWh generated is around 0.085 US$, and the initial investment cost is between 793 US$/kW and 2,427 US$/kW depending on location, cell technology and storage capacity ^13^. PV is a simple and modulated technology, that can be engineered, transported and installed easily in most of the regions of the world. This is a great advantage if compared to other renewable energy technologies such as geothermal, where a high degree of knowledge and technology must be applied, or if compared to wind power, where the barrier is not due to the technology per se, but the transportation of giant wind turbines seen as a logistic nightmare;

• *Concentrated solar power (CSP):* this technology uses the same source as PVs, the sun. Instead of taking advantage of its photons, it uses the thermal energy provided by solar irradiation concentrated on a small area to heat up a thermal energy carrier (usually salts, or water/steam) that will power a turbine to produce electricity ^17^. There are two main advantages of this technology when compared to PV ^77^: first, CSP experiences short term variations in cloudy days ^17^, and second, as a thermal process, heat can be easily stored for energy normalization purposes and to supply electricity during nighttime ^13^. There are four main CSP technologies that differ in how solar irradiation is gathered: solar power towers (SPT), parabolic through collector (PTC), linear Fresnel reflector (LFR), and parabolic dish systems (PDS), and one under development, concentrated solar thermoelectric (CST). We will not enter a detailed discussion for these technologies as it will not provide a contribution to this research. The performance of CSP depends on the amount of direct normal irradiance (DNI) provided in a certain geographical location, which intensity is greater between latitude 45˚S and latitude 45˚N ^17^, meaning that CSP could be globally available. In terms of land use, CSP requires a larger but irrelevant amount of land than PV technology for the same power output. On the negative side, despite installation costs declining on average by 28% on an annual basis ^13^, CSP’s CAPEX is still very high, standing between 3,400 $/kW and 7.000 $/kW ^13^. Moreover, its LCOE is also high if compared to other green technologies, standing at 0.185 $/kWh ^13^. The reason behind these high prices is due to the small installed capacity around the world (mostly Spain and the US) ^17^, which needed to be largely incremented to achieve a competitive financial structure. Last, even though CSP is a simple technology, as any new entry it may come with some technical challenges. Nevertheless, is very similar to PV, including its efficiency that achieves 18.1%, and many obstacles are overcome with the PV experience.

• *Wind:* approximately 2% of the total energy arriving from the sun to earth is transformed to kinetic energy into wind, and wind turbines convert this energy into electricity with no emissions ^78^. Wind energy is divided into two main sectors: onshore and offshore. The technology is the same, what changes are installation issues and finances. Even though wind energy is frequently present (especially offshore due to tidal winds), the real constrain of this technology is their sizes, as wind speeds grow with higher altitudes, obliging new generation of wind turbines to rise on average 86 meter from the ground ^78^, not only creating serious installation challenges, potentially creating fly zones constrains, killing birds flying by, but also changing the environmental sight around it. In terms of efficiency, even though the theoretical maximum efficiency of a wind turbine has been estimated to be 59% provided by the Betz Equation ^79^, this technology converts up to 50% to electricity with new generation turbines, a high rate but leaving little space for further developments. Cost wise:

◦ *Onshore*: like in PV cells, onshore wind power generation have seen prices dropping on average 13% on annual base, with today LCOE of 0.056 US$/kWh to produce and between 1,170 US$/kW (China) to 2030 US$/kW (UK) to install depending on location, storage capacity but above all, logistics ^13^.
◦ *Offshore*: for offshore wind power generation, logistics plays a key role in pricing this technology. Unfortunately, prices have dropped only 5% since 2010 ^13^, and CAPEX is yet very high, standing at 4,353 $/kW. The same is for LCOE, second only to CSP technology at 0.127 $/kWh.

• *Hydroelectric power:* this is probably the first renewable technology implemented by humankind, as it takes advantage of a simple process, the kinetic energy released by rain, which is accumulated by dams to spin turbines that produce electricity ^80^. Nevertheless, the implementation of this technology comes with some environmental issues, as dams usually permanently inundate large portion of the upstream territories in order to create the needed reservoirs. On another perspective, these reservoirs can stimulate local economies by providing water to the agriculture sector and be used as water sources for cities. Hydroelectric power is also very efficient, managing to transform up to 90% of its total energy potential into electricity ^81^. Nevertheless, this old technology requires thousands of hours of manpower, as it needs a strong engineering knowledge, long construction timeframes, large amounts of land works and the implementation can be very difficult. These installation challenges bring up the initial investment cost, which can be comparable to any other renewable energy source, between 1,193 US$/kW to 1,492 US$/kW ^13^. But the real advantage is the production price, which is the lowest among all renewables: 0.047 US$/kWh ^13^. Furthermore, this renewable source requires specific naturalistic traits such as rivers (with a certain water flow), availability of inundation areas, and piezometric jumps, which are limited traits in our environment. Most of the hydroelectric power sites in the world have already been located;

• *Biomass:* also known as biofuel, this renewable energy source is very controversial due to the fact that its feedstock may threaten biodiversity ^82^. Environmentalists are very concerned about the utterly use of this technology, as the plants grown for this purpose, such as switchgrass, willow trees and jatropha, not only damages the quality of the soil, but aren’t native in most of the world. Even the Food and Agriculture Organization of the United Nations (FAO) is very concerned about this issue and have produced a manual for policymakers named “Principles and Criteria for Sustainable Biofuel Production” ^83^. There are three possible energy carriers for which biomass can be transformed into: charcoal, oil or gas, using both thermochemical and biochemical conversion technologies ^84^. The principle is simple, convert the chemical energy stored in carbonaceous materials into a usable energy source. This process requires large amounts of arable land, large irrigation systems and special incentive policies, thus, not suitable for any territory. The initial investment cost is very low, between 950 US$/kW to 1,650 US$/kW depending on land price ^13^. The technology is mature, simple and not expensive, therefore has low installation challenges. Nevertheless, the production cost is more expensive than other renewable energy sources, averaging 0.062 US$/kWh due to its labor-intensive maintenance nature ^13^. In terms of efficiency, according to the Biomass Energy Resource Center ^85^, transforming biomass into electricity will provide a range of efficiency between 20% and 25%, low if compared to other similar resources. Converting municipality waste into energy is also considered to be a biomass process, but it depends on human activities for its feedstock, not from a natural replenished, and therefore will not be considered in this research;

• *Geothermal:* this technology was first used to produce electricity in Lardarello field in Italy in 1913 ^86^. The physics is very simple, to reach and use underground steam reservoirs (hydrothermal), heated by the earth’s core, to spin steam turbines to produce electricity ^87^. The difficult part is not the physics, but the drilling. Earth’s core heats our crust uniformly, but in some specific areas with certain geological characteristics, the heat reaches the surface. When this heat encounters a water reservoir, the water may surface as steam (Geysers), as simple heated water (hot springs), or be trapped underground (steam reservoirs). Drilling at these temperatures may be a challenge, which spikes the initial investment cost today being between 2,500 US$/kW ^86^ and 3,976 US$/kW ^13^. Maintenance of the equipment may also be costly, as shown in the electricity production price of 0.072 US$/kWh ^13^. The minimum temperature required for the water reservoir to sustain an electricity geothermal power plant is approximately 150C° ^86^. According to the temperature of the reservoir, three different technologies may apply: dry steam, flash steam or binary cycle. Unfortunately, sometimes the heat coming from Earth’s core near the surface do not encounter a water reservoir and ends up heating the rock. When this happens, a technology called enhanced geothermal system (EGS) must be used. EGS is a process in which fluids are controlled-pumped underground to reactivate pre-existing fractures and restore permeability ^88^. This allows to expand geothermal sites and geothermal potentials. Nevertheless, efficiency may be a problem, as the geothermal-to-electricity process manages to convert on world average only 12% of steam power into electricity ^89^. The good news is that geothermal power plants require small acreages ^86^, as most of the work is done underground. The geothermal-to-electricity process cannot be confused with geothermal heat pumps (GHP), or ground source heat pumps (GSHP), as these are used only to heat up buildings or water, but the temperature of the fluid does not allow for electricity production. A GHP or GSHP system can be installed basically anywhere in the world, as temperature requirements are much lower.

• *Oceanic:* there are four major types of processes today that can convert the energy accumulated by the ocean into electricity, three of them converting kinetic energy from tides, waves and currents, and the other converting ocean thermal energy (OTEC) from the ocean temperature differences ^90^. The first three technologies use the motion of the water (waves, tides or currents) to spin turbines and produce electricity through buoys, floaters, and other submergible or floating devices. The OTEC technology uses a different approach, taking advantage of the difference in temperature between the surface of the ocean, exposed to sunrays, and the cold-water temperatures at over 1,000 m deep. The difference of only 20°C can yield a positive result ^90^. OTEC can be implemented using two different processes: a closed cycle that uses a working fluid such as ammonia, and an open cycle that vaporizes surface water in a vacuum. The choice between these processes will be according to location, sizes, water temperature at the surface, and other factors. In Burman and Walker ^90^ work, prepared for the US Department of Energy, they sustain that the major challenges of this technology are due to its nature, the ocean. First, the environmental impact can be a concern, as by developing such structure on water there is the need to prioritize the ocean biodiversity and ecosystem and be sure to impact the least. Consequently, this brings us to the second challenge, obtaining site permits. Permits are released not only based on the environmental study, but also based on transportation routes, national security issues, and many other factors. Last, this technology faces the dilemma of being far from any electrical grid. The cost to transport the electricity produced and injected into a national grid, may be more expensive than the technology itself. On the other hand, what is interesting about this technology is its efficiency rate that stands between 70% and 90% due to the water density ^57^. Unfortunately, this technology has not been implemented much, therefore it’s hard to estimate a correct value for electricity production and initial investment cost. There are some infrastructures around the world such as Sihwa tidal power station (South Korea), La Rance tidal power station (France), Annapolis Royal generating station (Canada), Jiangxia tidal power station (China), Kislaya Guba tidal power station (Russia), with massive differences between each other in terms of their investment and production costs ^91^. Salinity gradient technology is also considered to be an oceanic energy source, as it takes advantage of the difference in salinity at river mouths. This study will not consider salinity gradient due to its small practical use. 

**Table 2.** Renewable energy sources’ main traits.

	CAPEX $/kW	LCOE $/kW	Efficiency	Installation Challenges	Geographical Availability	Land Requirements
**PV**	793 – 2,427	0.085	15% – 30%	Very Low	Very High	High
**CSP**	3,400 – 7,000	0.185	17.3% – 18.1%	Medium	Very High	Medium
**Wind** **• Onshore** **• Offshore**	1,170 – 2,030 4,353	0.056 0.127	Up to 50% Up to 50%	High High	High High	High High
**Hydroelectric**	1,193 – 1,492	0.047	Up to 90%	Very High	Low	Very High
**Biomass**	950 – 1,650	0.062	20% – 25%	Low	High	High
**Geothermal**	2,500 – 3,976	0.072	Up to 12%	High	Very Low	Low
**Oceanic**	Uncertain	Uncertain	70% – 90%	High	Medium	Very Low
	**Financial**		**Technical**		**Environmental**	

We finally arrived at the last piece of our SURESC model, the source selection. We have explored the different and most common renewable energy sources exploited on our planet, now we need to select what best could fit our upstream SURESC model. Table 2 resumes all major traits of these energy sources, and some considerations must be done. If the goal of the SURESC model is to diminish time to achieve price parity between green and brown energies by selecting one technology where to address resources to, even if in different situations different sources are superior (Norway, Brazil and Colombia for hydropower), we need to select a widely distributed energy source with high expansion capabilities. As stated, hydropower is geographically restricted, and most of its potentials are well known or well exploited ^92^. Shayegh *et al.* ^42^ show that hydropower learning curve is stagnant, thus investing resources into it would potentially mean to sustain the hydropower industry for a very long period of time. Consequently, we can exclude hydropower form our SURESC model. Next to be dismissed will be geothermal, which apart from being available only in regions where the core heats the crust, its efficiency is very low and its CAPEX very high. Iceland is doing a good job with this technology but has unique naturalistic traits that allows for a feasible usage of geothermal. Oceanic energy instead, is available globally but unavailable to all landlocked countries, risking an energy dependence from coastal neighbors. Moreover, this technology is at early stages (not mature), where CAPEX and LCOE haven’t reached a stabilized value. For it has to be dismissed too. On another level, as mentioned previously, biomass is a very controversial argument among the energy sector, as its implementation may threaten biodiversity ^82^, thus will be dismissed too. We are now left with three mature renewable technologies, PV, CSP and wind. Not accounting for offshore wind farming due to its high CAPEX and high LCOE, onshore wind energy has also its issues, as winds tend to be stronger at higher altitudes and therefore turbines must rise very high from the ground ^78^, creating an arguable ugly visual impact, strong logistics constrains, environmental issues (killing birds, mainly raptors) and creating aerospace security concerns. Nevertheless, it has the lowest LCOE among all renewable energies, possess an acceptable efficiency and can be delivered widely. But “solar energy dwarfs all other renewable and fossil-based energy resources combined” [ 17, p.467], compensating for its low efficiency rate provided by both PV and CSP, and can potentially produce sufficient energy to supply long-term growth. Consequently, I believe that solar energy would be preferred over wind energy on a global scale. We are now left with the last two: photovoltaic and concentrated solar power. This is a hard choice, as both are very similar, and more studies comparing these two technologies should be done. At status quo, I am obliged to choose PV over CSP for only one reason: costs. This seems unfair, as CSP has not seen the same technological evolution as PV, and with time, it could become a strong PV competitor, but today is too expensive both in CAPEX and LCOE and must be discarded. Finally, we have the last piece of our puzzle, solar PV, which will be the choice for our upstream SURESC model.

## Conclusion

The main trait of the SURESC model is to limit the number of technologies receiving resources, allocating more resources to less technologies and speed-up technological price parity with the competition. In this paper I have proposed a possible implementation of the SURESC Model, but to extend our analyses on hydrogen, the International Renewable Energy Agency (IRENA) has produce a report prepared for the 2 ^nd^ Hydrogen Energy Ministerial Meeting in Japan ^64^, which updates hydrogen’s role in the renewable energy sector. Their findings strongly suggest that hydrogen should have a central role in the decarbonization of power production and hydrogen produced from renewable sources could become competitive soon. Furthermore, IRENA suggests that hydrogen can be used as a downstream energy provider for vehicles and heat power, in symbiosis with fuel cells. Even though there are some initiatives in this direction ^93, 94^, I don’t believe this will be a market changer, and I strongly discourage the pursue of this strategy for three main reasons:

1.   Hydrogen is a very flammable element and must be handled with care. To diminish risks associated to human safety, the environment and to things, hydrogen should be produced and embedded into ammonia in a confined and secured space. Ammonia is safer to handle and doesn’t ignite so easily.

2.   Electric powered vehicles are already on the marker. Tesla, as a pioneer, is gaining daily market shares in the automotive industry, which encouraged all major automotive makers to follow Tesla’s example. Volvo, Toyota, FIAT, Renault, BMW, Porche, Mercedes-Benz and others, all have hybrid or electric motors options associated to their models. Countries all over the world are implementing strong incentives for owners to buy electric cars. Creating a new hydrogen market didn’t worked out in the past, and just won’t be competitive anymore.

3.   Creating a new distribution network for hydrogen-fueled vehicles would cost much more than to adjust the current electrical backbones to provide electrical energy dispensers. The same principle can be applied for home use of hydrogen as a fuel.

Even if many studies suggest that hydrogen could potentially substitute natural gas in existing middle stream infrastructures ^69– 73^, but implying that such conversions require a case-by-case analyses rather than a general assumption, many others point to ammonia as a preferred energy carrier over hydrogen ^55, 59, 67^ for existing infrastructures. 

As a final observation, we need to underline that the two key elements to reduce the price of ammonia are the costs of electrolyzers (used in hydrogen production) and the LCOE derived from solar PV ^13^. Electrolyzers are scaling up quickly, form MW to GW, but their prices are decreasing slowly, and If no breakthrough comes along, projections show a reduction on half of today’s price (840 $/kW) by 2050 ^13^. This trend is too slow, and incentives towards decreasing costs of electrolyzers at a faster pace should be taken into serious consideration. Diverting part of the resources used today in other technologies could incentive a faster price reduction. PVs’ LCOE depends on the scale of the market and technological advancements (R&D), but even with todays’ technology, PV could potentially provide a competitive LCOE if PV farming grew in numbers. As a real-life example, Saudi Arabia intends to start producing green ammonia form a hybrid wind-PV system as an alternative fuel for their power plants’ feedstock ^13^. The delivery price with today’s technology is forecasted to be on average at 5 $/kg of ammonia, and according to Nagashima ^95^, parity with fossil fuels would be achieved in the country with a price of 3.5 $/kg (-42%). This is very encouraging, as Saudi Arabia is the 3 ^rd^ largest world oil producer, where its oil extraction price is very low, making it a good fit for top benchmark price parity between green ammonia and fossil fuels. Therefore, in other countries this parity should be achievable with a smaller coefficient.

**Figure 11.**
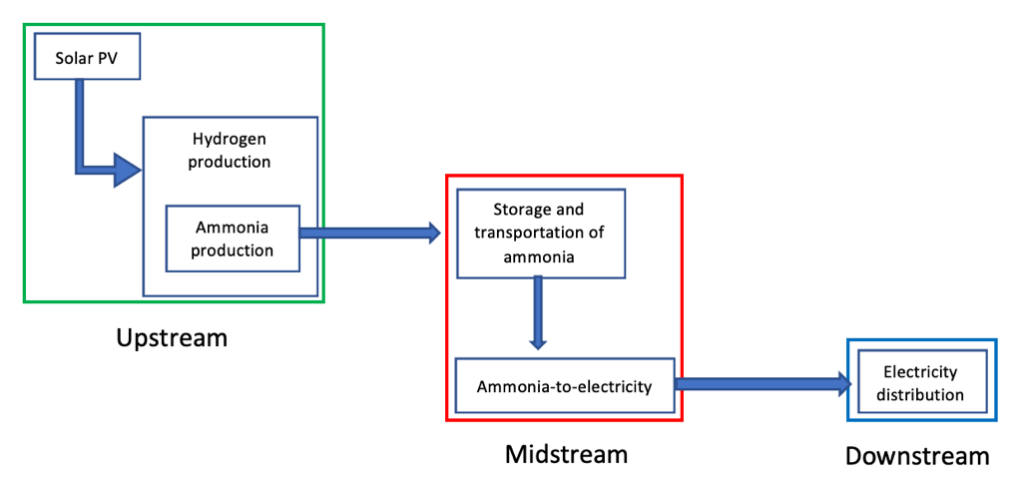
Possible implementation of a SURESC model.

Based on the analyses above, Figure 11 indicates what a SURESC model could possibly be like.

## Recommendations

The SURESC model per se will speed-up the energy transformation process but it may not be enough, as it requires governments coordination with coordinated legislations ^39^ creating non-convex economies with convenience equilibria through R&D and taxes/subsidies. Even though standard economic framework suggests that markets and not policymakers and energy geeks should decide winners and losers, in order to speed-up the energy shift process, governments should incentive a technological restrictive resources allocation policy to achieve a roadmap designed to implement the SURESC model. Furthermore, investments in new oil and gas explorations and the development of new certified reservoirs should be heavily discouraged. In 2019, new investments made on the oil and gas upstream only summed up at an astonishing 505B$, compared to 166B$ of subsidies globally allocated to the renewable energy upstream industry, and keeps rising at an average of 4% on a year base ^36^. If that money would have been invested on a clear road map, SURESC could play an important role and the energy shift could be a reality in a relatively very short period of time. The issue is not to mitigate new emissions, but to diminish the existing ones and contemporarily quench new energy needs, as we are already producing more CO _2_ than the earth can possibly absorb. Perhaps, a possibility would be to pair the SURESC model to the ineffective carbon credits ^96^ formalized by the Kyoto protocols of 1997, and relaunch them as a new green strategy. This opens the opportunity to new research topics.

### Limitations and future studies

As a preliminary study, this research is intended to provide a high-level analysis on the renewable industry, consequently not digging enough into each technology, and further studies are required as it presents few limitations and new research opportunities:

1.   A more in-depth study on each SURESC’s stream is required to check that figures and numbers align.

2.   This research has considered only tested technologies and dismissed those in a conception phase, which in the future could grant a better overall efficiency and become a technological breakthrough;

3.   Even if there is plenty of literature on solar PV applications, the environmental impact of producing and installing solar panels on such a vast scale needs to be addressed, so those of a large-scale electrolysis for hydrogen production. Other issues related to this industry such as rare earth exploitation needs further studies and discussions;

4.   The renewable industry is very dynamic, where prices change on a monthly base, therefore there is a need to monitor the evolution and constantly update numbers;

5.   The geopolitical implications of such an energy shift are not properly approached, issues like an energy free market or energy dependences require a better understanding and further studies;

6.   More studies on the consequences (both to the environment and to humans) for hydrogen, ammonia and NO2 leaks into the atmosphere should be done;

7.   There is the need to quantify SURESC in terms of costs, and size it respectively. 

## Data availability

All data underlying the results are available as part of the article and no additional source data are required.
