# Standardizing a unique renewable energy supply chain: the SURESC Model

**DOI:** 10.12688/f1000research.27345.3

**Published:** 2021-11-15

**Authors:** Emiliano Finocchi

**Affiliations:** 1 Temple University, Rome, Italy

**Keywords:** Research and Development, Carbon-free Technology, Energy Policy, Energy Shift, Renewable Energy, Energy Standards, Learning Curve

## Abstract

This theory-building research intends to dig into the renewable energy industry and drawing from research on learning curves and energy polices, proposes a way to speed-up the energy shift from our fossil-fuel dependency to a green economy. Even though standard economic frameworks suggest that markets and not policy makers should decide winners and losers, we urge to accelerate renewable energy competitiveness, proposing that by limiting the number of maturing renewable technologies where resources are allocated to at government level, we reduce the time within which renewables will achieve technological price parity with fossil fuels. In turn, by analysing the energy demand and supply curves, the study suggests that this action will also mediate the relation between quantity and price, shifting only the supply curve, leaving the demand curve unaffected. It continues by proposing the *standardization of a unique renewable energy supply chain* model, defined as the SURESC model, relating the indirect effect of limiting the number of maturing technologies to allocate resources, to achieve renewable price-parity with conventional energy sources faster. This is a preliminary theoretical study intended to provide a holistic approach to a known problem.

## Introduction

### Brown energy and dependency

“Despite the growing use of commercial energy, the world faced very considerable fuel poverty. Many remain without access to electricity and to modern cooking fuels” [ 1, p. 3].

**Figure 1.**
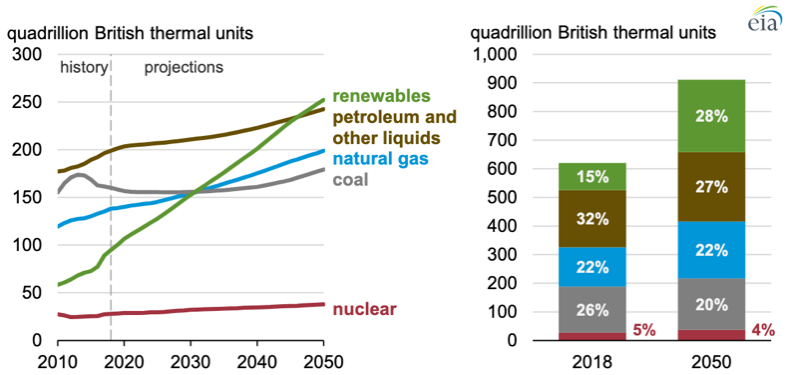
Global Primary Energy Consumption by Energy Source. (Source: U.S. Energy Information Administration, 2019.)

In the world’s geopolitical puzzle, energy plays a key role defining each piece and a driver to seek for global prosperity and security ^2^. Not all countries produce enough energy to impact the world's geopolitical scenario, but the global energy industry affects the geopolitical interests of all countries ^3^. In fact, for every 10 percent of oil price reduction, the world’s GDP grows 0.2 percent ^4^. As seen in Figure 1 ^5^, fuel such as petroleum and coal (fossil-fuels), account for more than half of the entire energy consumption source today, making it a strategic commodity for geopolitical bargains.

Forecasts for the near future do not seem to foresee any change, as even though renewable energy will largely increase by 150% by 2050, energy consumption will also increase by 50% driven by the Asian market ^5^, thus by 2050 petroleum and coal will still be accounted for 47% of the entire energy consumption source ( Figure 1). We need to change this trend urgently!

Not least, the impact to the environment for such amounts of fossil-fuel consumptions is well documented ^6– 9^ and is responsible for today’s climate change ^9^. In fact, Höök and Tang ^9^ suggested that as fossil energy and climate change are strongly correlated, a solution must be found by treating them as “interwoven challenges necessitating a holistic solution” (p.1). Considering Höök and Tang ^9^ work, this study intends to develop a holistic approach towards this well-known phenomenon, and underlines the importance of a common, standardized eco-friendly solution where all major resources should be canalized in order to obtain a faster energy shift (from fossil to renewable) than forecasted today. As we will discuss, no solution is perfect, but we need to balance pros and cons prioritizing global warming mitigation processes, which is one of our major responsibilities to guarantee a sustainable future.

### Dangers of unbalancing the energy geopolitics

“Even with renewable energy and energy efficiency, markets and not goodwill to arrest climate change, largely determine the path of investment” ^3^.

Even though renewable energy technologies are an extremely attractive energy replacement, as we will determine later in this research, fossil-fuels are hard to die. Business leads the way, and we will require a degree of human resources management similar to the one used in the cold war when this shift will happen ^3^. The uneven distribution of the world’s natural resources causes great inequalities and attract states and private organizations to aspire access grants to resources in foreign territories ^10^. In fact, even though many international agreements on renewable energies have been signed widely (e.g., UN Framework Convention on Climate Change, Kyoto Protocol, Paris Treaty), only few countries seem to be on track for a long-term total decarbonization strategy. Europe (27 countries) is a positive example, as with its Green Deal intends to achieve zero emissions by 2050. However, countries like the US alone have passed from being the 3 ^rd^ biggest oil producer in 2008, to be the biggest oil producer in the world in 2019 ^11^, nearly doubling its production in 10 years.

### The need for a standardized energy stream model

“The disrupting elements of rapid change can be mitigated by common goals and a clear roadmap where incumbents join new players in implementing a low-carbon global energy transformation roadmap” [ 12, p. 20].

In the past years we have seen new renewable technologies and new renewable concepts rising at an astonishing rate. Today, we are producing (or potentially produce) green energy from wind, solar (PV and CSP), hydropower, biomass, geothermal, oceanic (tidal and current), cellulosic ethanol, artificial photosynthesis ^13^ and more, and yet, none of these technologies have reached fossil-fuel price parity.

The allocation of resources to realize these countless renewable technologies are massive , each with standalone projects (most of which are immerged in regional realities), lacking a holistic vision ^9^ to boost-up and turn into the leading energy feedstock of the future. Standardization could be an important part of this process but is dependent on the maturity of the technology. Quebec’s Normalization Bureau ^14^ define standards as a set of agreements among players of a given industry defining characteristics and rules tailormade for that industry using benchmarks from field’s collective knowledge. Standardization means to understand and approach a problem in an agreed way (as a voluntary action), based on field knowledge and studies, and can leverage an innovation journey that can lead to excellence ^15^. Many studies on standardization have shown how standards are important to enhance the development of the industry involved ^14^, from medicine ^16– 18^, to HR ^19^, to Oil&Gas ^20^, to IT ^21, 22^, all industries have standardized themselves with time. Moreover, standardization diffuses knowledge, increases predictability and reduces uncertainty and risks ^15^, key strategic factors for small and medium enterprises (SME).

The energy sector is extremely complex, data is sometimes not available ^23^ and economic models depend on geopolitics, R&D advancements, competitive alternatives, subsidies and/or taxes, externalities, industry’s supply and demand elasticity and other factors that rapidly change with time ^24^, thus, are hard to determine. Moreover, the energy sector is heavily politicized, where usually the fossil fuel supply chain is subsidized (from supplier’s governments) and taxed (from demander’s governments) whereas green energy is generally subsidized to help it achieve competitive traits ^25^. Policy makers are trying to incentivize a green economy, but we are far from a real shift, as most forecasts have been missed, and we continue to increment the global usage of carbon energy despite all efforts. In 2017, total subsidies to the renewable energy upstream touched 166B$ globally (between private and public) ^26^, with an increment of 7.09% production from the previous year ^27^, equivalent to 442 TWh of extra power generated, and forecasts aim at 192B$ subsidies for 2030 ^26^. In the energy context these numbers aren’t even close to sustain an energy shift, as in 2017 global energy demand grew 543 TWh from 2016 ^28^ driven by the Asian markets, yet renewables alone couldn’t cover this need, lacking 101 TWh of power generation that has been supplied by carbon fuels. As suggested by the US Energy Information Administration ^29^, by following this trend our carbon emissions will continue to rise rather than fall, and our carbon dependency will be maintained ^3, 5, 10^. Furthermore, standard economic framework suggests policymakers to apply policies seeking for price parity in order to achieve competitiveness among energy sources. Nevertheless, price parity between green and brown energy may not be enough if economic models don’t account for fossil fuel price response to renewables, as governments producers of fossil fuel may use their price buffer created from taxes and royalties as a response to push fossil fuel prices yet lower ^23^. This suggests that to shift to a green economy, renewable’s price target should aim lower than the current fossil fuel benchmark and its economic models should foresee a strong economic response from its competitors. Technocrats and economists have spent thousands of hours creating models that would incentivize renewable energy deployment, and despite all our knowledge and efforts, we are behind in our green workplan, and our planet seems to be worming up faster than predicted ^30^. Global warming puts our external models (social, environmental, economic, etc.) under a large dose of stress which will consequently unbalance these systems. In non-stressful situations, it would be correct to leave to markets and time the burden of a “natural selection” of technologies, but in today’s environmental pressure, we can’t afford to wait for a technological breakthrough anymore, governments should model conventional economies with current technologies and regulations to create non-convex economies with convenience equilibria through R&D and taxes/subsidies. We need to act by maximizing all resources and create a coordinate globalized network of protocols that will help to speed-up this shifting process. This concept is not new to the literature and can be partially found in the Carbon Leakage theory ^31^, which states that in the absence of a global coordinated climate change policy, industries relying on fossil fuels as their primary energy source may relocate their premises in countries with less restrictive policies.

“Extremis malis extrema remedia” (Latin saying). The literature seems to point towards a mix use of renewable energies solution ^9, 12, 32– 35^, and even though standard economic framework suggests that markets and not policymakers and energy geeks (like myself) should decide winners and losers, I propose that there is a direct positive relation between the number of renewable technologies subsidized at the government level and the speed with which these technologies can achieve price parity with fossil fuels. In other words, the greater the number of renewable technologies our governments subsidize, the more we increment the time within which these technologies will take to reach price parity with fossil fuels. Diminishing the number of renewable technologies to subsidize will increase resource allocation to selected technologies which consequently will speed-up its deployment and price parity achievement.

## Limiting the number of technologies

“Governments have been very creative in imposing price control, what is needed is to show the economic costs of those actions and evaluate fewer damaging alternatives” [ 36, p. 675].

It is important to underline that this is a theory-building research ^37^, and not an empirical study. The intent is to provide a theoretical framework that may serve as a base for future empirical studies.

Learning curves play an important role to understand maturing industries such as renewables, as the sizes of the investment needed for a certain renewable technology to reach technological price parity (leaning investment) with fossil fuels may define the time to achieve this goal ^38^. In this context, research and development (R&D) can diminish learning-by-doing investments in different ways ^39^, pushing the learning curve down by curve-shifting or curve-following (for more references on curve-shifting and curve-following please consult ^38^), diminishing the learning investment. Even R&D innovation catch-up could act as a catalyser but requires continuous efforts through the establishment and adequate support of competence-creating units ^40^.

**Figure 2.**
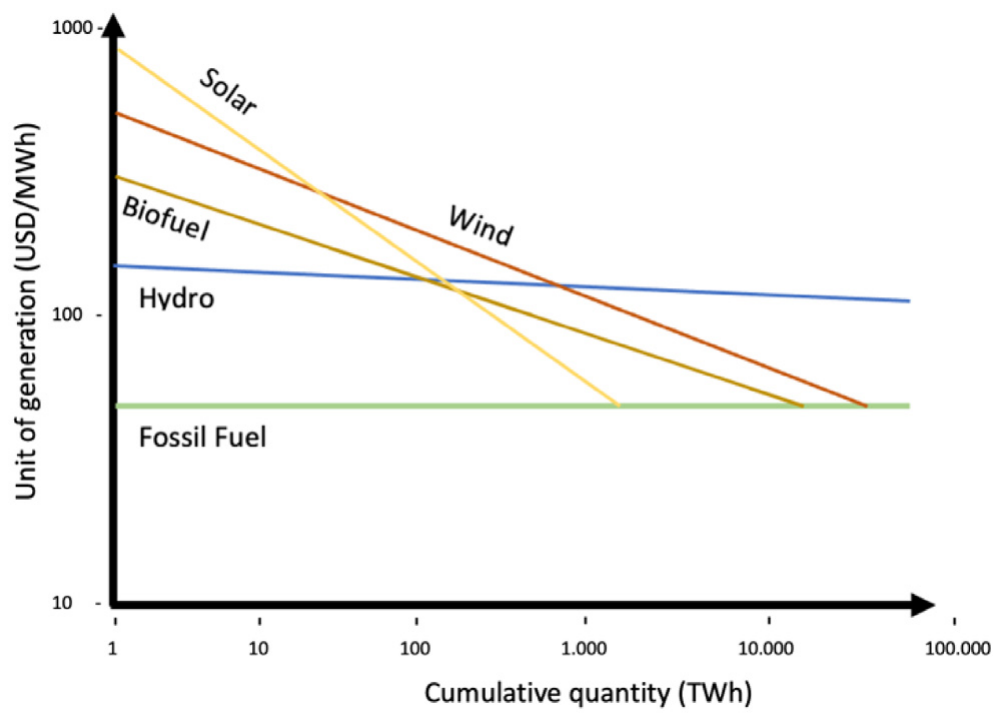
Green technologies learning curves based on learning-by-doing.

**Figure 3.**
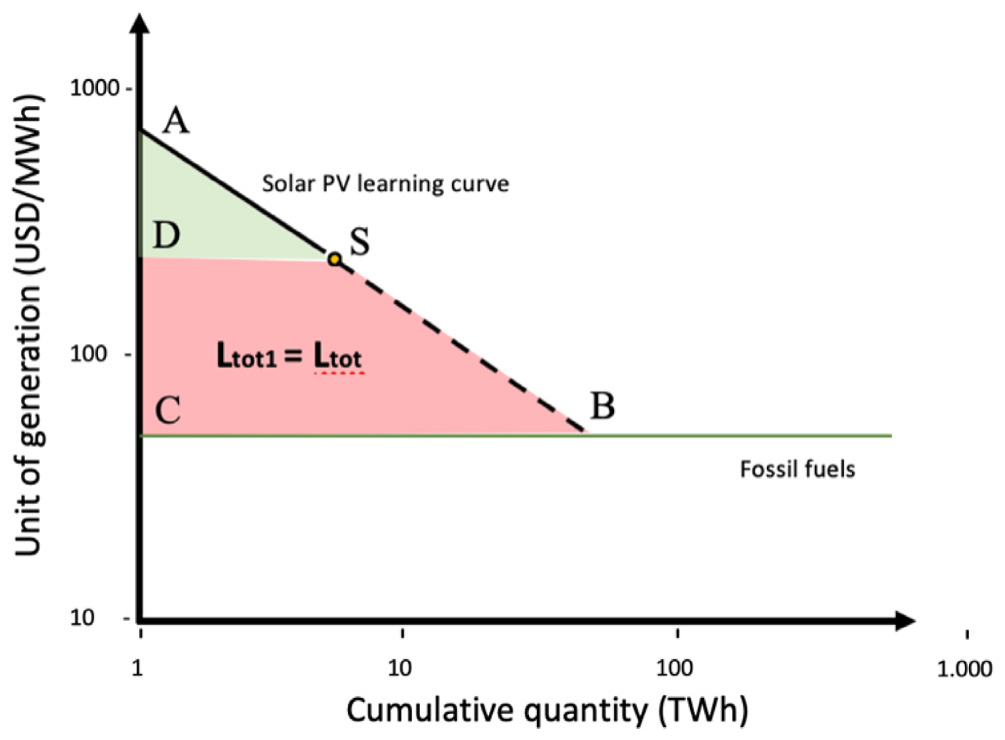
Solar PV learning curve based on learning-by-doing.

Expanding on Shayegh *et al.* ^38^ learning curve for Solar PV on a learning-by-doing bases (without R&D) represented in Figure 2. Fossil fuel unit cost of generation has been set at a fixed price of 50 USD/MWh, supposing it has reached it maximum learning point where cumulative quantity will not affect unit cost (benchmark). Solar PV has a learning quotient of 23%, intercepting fossil fuel line (price parity) with a future cumulative quantity (power deployed) at over 1000 TW. On the Solar PV line, the continuous line represents historical data for Solar PV technology, the dot represents status quo, and the dotted line after the dot represent forecasts. The area between the Solar PV line (in all its length) and the fossil fuel line in Figure 3 ( $\bar{\mathrm{ABC}}$ ) is the total amount of learning investment needed to reach price parity. The area within the dotted line and the fossil fuel line represents what yet needs to be invested ( **Ltot1** = the difference between the total amount to be invested $\bar{\mathrm{ABC}}$ and the total amount invested so far $\bar{\mathrm{ASD}}$ ):

$$\bar{\mathrm{ABC}}-\bar{\mathrm{ASD}}=\bar{\mathrm{SBCD}}=Ltot1=\text{Ltot}$$

$\bar{\mathrm{ABC}}$ = Total learning investment to reach price parity

$\bar{\mathrm{ASD}}$ = Total learning investment historically allocated

$\bar{\mathrm{SBCD}}$ **, Ltot1** = Total learning investment that needs allocation

**Ltot =** Sum of the total learning investments among selected technologies that needs allocation (only Solar PV, **Ltot = Ltot1**)

**Figure 4.**
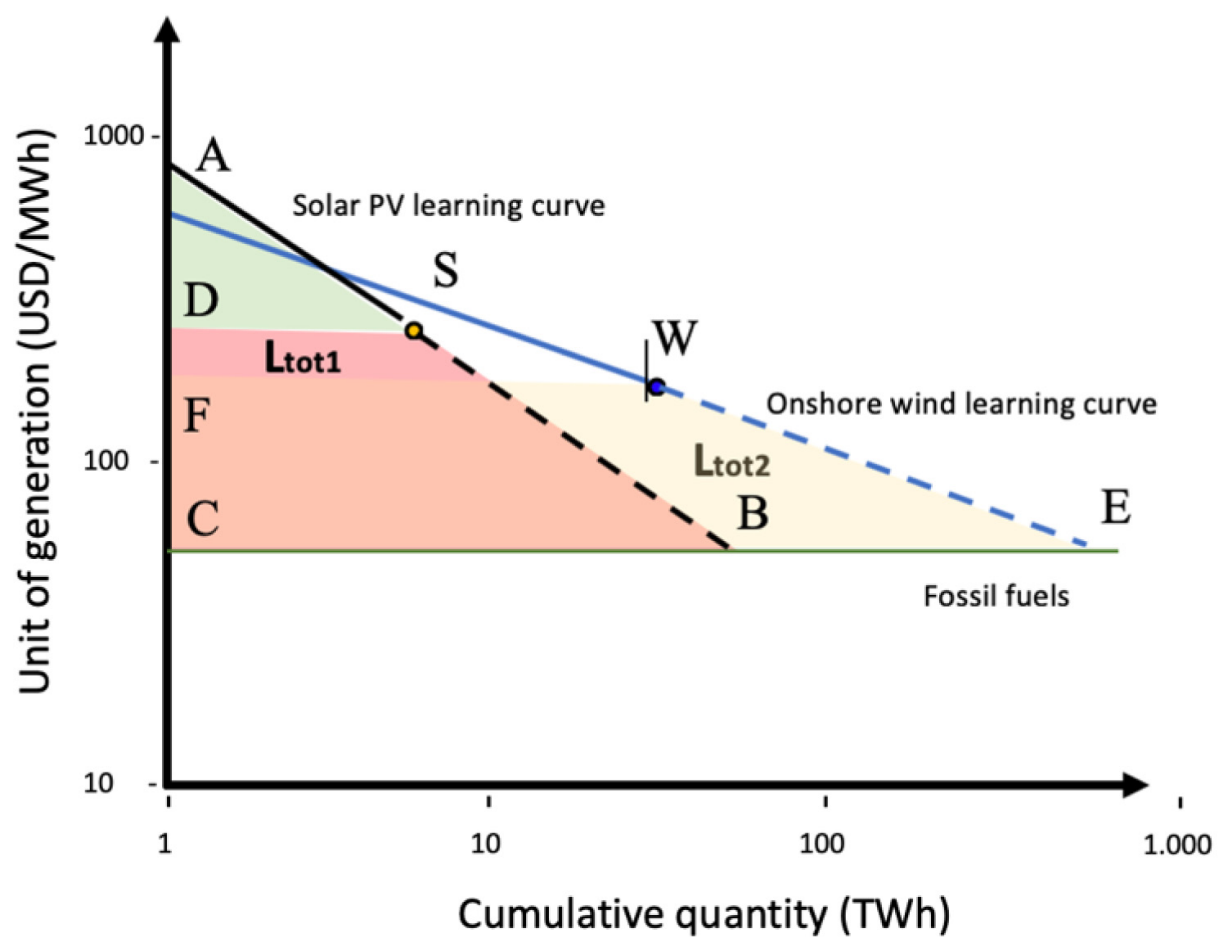
Solar PV and onshore wind learning curve based on learning-by-doing.

Now we introduce a second renewable technology, onshore wind ( Figure 4):

$$\bar{\mathrm{WECF}}=\text{Ltot2}$$

Ltot1+Ltot2=Ltot

By introducing **n** technologies, we have:

Ltot1+Ltot2+Ltot3+...+Ltotn=Ltot

Setting **Ltot1** as the smaller area, we have **Ltot2** = **Ltot1** + C1, where C1 is the difference between **Ltot2** and **Ltot1** ( **Ltot2** – **Ltot1**).

Therefore,

Ltot1+(Ltot1+C1)+(Ltot1+C2)+...+(Ltot1+Cn-1)=Ltot

$$\text{nLtot1}+\sum_{n-1}^{n-1}\mathrm{Cn}\;=\;\text{Ltot}$$

$\sum_{n-1}^{n-1}\mathrm{Cn}\;$ represent the sum of the area differences between the area $\bar{\mathrm{SBCD}}$ (solar PV learning investment needed) and the other renewable technologies areas, and therefore is a constant and can be simplified as **Ctot**. ***n*** represents the number of technologies.

nLtot1+Ctot=Ltot

*Ceteris paribus* but **n** and **Ltot**, so that **Ltot** becomes a function of **n**:


Ltot=Ltot1n+Ctot

Where **Ctot > 0**, **Ltot1 > 0** and **n > 0**.

This function represents the total amount of learning investment required for a set of renewable energy technologies to reach fossil fuels price parity. Usually, learning curves are indeed curves and not straight lines, but to the purpose of this exercise, the result would not change.

Continuing; every year both the private sector and the public sector allocate finite resources into the learning investment of renewable sources. These resources can be in the form of equity, subsidies, credit, loans, studies, etc. and are finite in nature (166B$ only in 2017’s subsidies as per Tylor ^26^). To simplify our calculations, let’s idealize a *constant* yearly resources allocation ( **RA**) to the learning investment **Inv**, and an equilibrium in the distribution of resources among technologies. This means that, every year ( **t**), the total learning investment **Ltot** required to achieve price parity decreases of a value of **RA**.

Ltot(t1)=Ltot(t0)-RA; Ltot(t2)=Ltot(t1)-RA;...;Ltot(tz*)=Ltot(tz*-1)-RA

$$\;*\text{z}=\frac{\mathrm{Ltot}}{RA}$$

**Figure 5.**
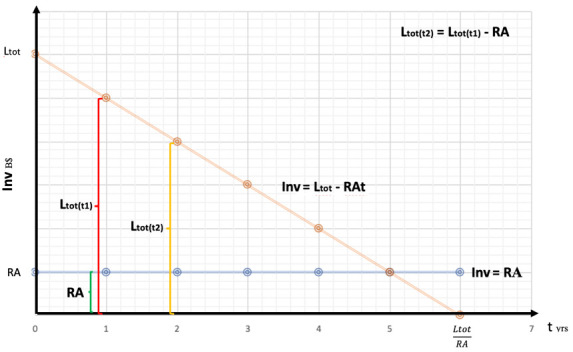
Effect of resources allocation into a single technology.

On the **Inv** axes of Figure 5, we have the amounts of resources (manhours, subsidies, quantity control protocols, etc.) converted into equivalent B$ as a function of time (t). The **RA** line represents the constant resources allocated year by year to the renewable industry by the private and public sector.

In Figure 5 we can see the amount of possible **RA** converted in USD as a function of time:

Inv=Ltot−RAt

We now substitute **Ltot**:


Inv=Ltot1n+Ctot−RAt

Price parity will be achieved when **Inv** = 0, this means when $\text{t}=\frac{\mathrm{Ltot}}{\mathrm{RA}},$ and by substituting **Ltot**:

$$\text{t}=\frac{\mathrm{Ltot}1n+\mathrm{Ctot}}{\mathrm{RA}}$$

As we can see, on a learning-by-doing process, as the number of technologies requiring learning investment increase, time to achieve price parity increases too. There is a direct, positive relation between time and the number of technologies where resources are allocated. Investing into R&D would shift the learning curve down, acting as a negative moderator between **t** and **n**. This means that as the sum invested into R&D increases, both time and learning investment decrease. Let’s demonstrate this claim:

Following Shayegh *et al.* ^38^ research, we find that investing in R&D will diminish initial investment costs **Ltot** and will push our learning curve down (for both curve-following and curve-shifting). In our model, it means that we diminish the initial **Ltot** by an **X** amount provided by the R&D process resulting into a new **R&DLtot** ^20^. Thus:

R&DLtot=Ltot−X

We now substitute **Ltot** with **R&DLtot:**

$$t=\frac{\mathrm{Ltot}1n+\mathrm{Ctot}-X}{\mathrm{RA}}$$

**Figure 6.**
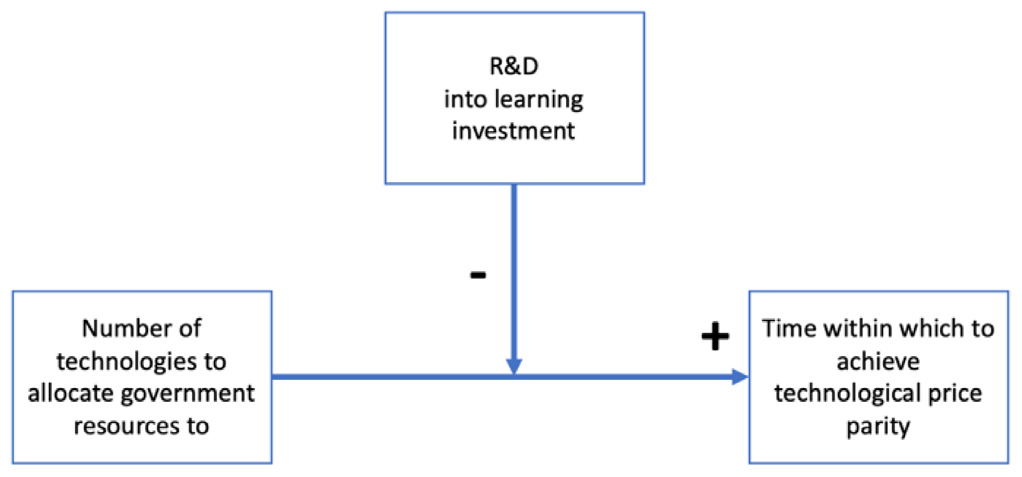
Model of the importance of resources allocation to specific technologies.

As **t** is a function of **n** (t(n)), **X** will act as a negative moderator to this function ( Figure 6). As **n** is a positive integer greater than 0, to minimize **t**, we should allocate resources to R&D for **n=1**.

Considering that we are discussing at government level, another obvious result shown in this equation is that time is negatively related to the size of the resources allocated **RA**. This means that as more resources governments allocate, faster will parity price be accomplished (and vice versa).

We have found evidence to support that technological price parity between renewables and fossil fuels will be reached faster if limited government resources would be allocated to a restricted number of technologies. Reaching technological price parity means decreasing unit cost of generation price and increasing cumulative quantities too. We now need to apply this find to demand and supply renewable energy curves and see how the market would react by increasing and decreasing the number of technologies **n** we allocate resources to. Renewables produce electricity; thus, renewables’ commodity is electricity. General demand and supply linear curves in an *ideal market* are represented by the following inverted functions for both demand and supply:

- Demand: **P = a - bQd**
- Supply: **P = α + βQs**

*Ideal markets* require perfect competition, property rights well established, information availability, low externalities and no decreasing average costs as production increases ^24^. We will adapt from Dahl’s ^24^ coal demand and supply curves to electricity as main commodity, where in ideal markets the author describes **b** and **β** as the slope (elasticity), and **a** and **α** as the sum of the following parameters:

*Demand* **b**

- Price of substitutes to electricity (-Pse), such as natural gas used in stoves rather than electric stoves;
- Price of complements to the commodity (Pce), such as electric heaters vs gas heaters;
- Price for the technology for electricity use (+/-Tcu), such as electric cars vs diesel cars. This parameter may be positive or negative depending if the electric technology is cheaper than the alternative technology;
- Price of the output produced (-Pop). Prices of services using electricity as feedstock such as fun-parks, electric go-karts, and others;
- Energy policy (+/-Pol). It depends on the policies in place, positive for subsidies and negative for taxes;
- Number of buyers (-#buy)

*P=(Pse−Pce +/-Tcu+Pop+/-Pol+#buy)−b(Qd)

*Inverted curve: **Qd** is the demand quantity and **P** is the price

Parameters in the demand curve has no direct effect with the production technology. Demanders only know supplier’s selling price. The demand curve will not be altered by altering **n**.

*Supply* **β**

- Price of factors for producing electricity, such as labor and capital (-Pf);
- Price of similar goods that power plants could produce (-Psim). If oxygen or chlorine prices increase to a point where using electricity for electrolysis would provide more profit than selling electricity directly, green power plants could be enticed to change production;
- Price of by-products or complements of electricity production (Pb), which in renewables do not apply;
- Production technology (Tc), technical changes should reduce costs and increase production;
- government coal policies (+/-Pol), they could incentive or disincentive the industry;
- the number of sellers (#sell);

*P=(-Pf−Psim+Tc+Pb+/-Pol+#sell)+b(Qs)

*Inverted curve: **Qs** is the supply quantity and **P** is the price

**Tc** represent the technical changes throughout time; the technological advancement pace of a specific technology that will determine a cost reduction and an increase in production.

$$\text{Tc}=\frac{\text{Number}\;\text{of}\;\text{Technical}\;\text{Changes}}{\text{Time}}$$

**Figure 7.**
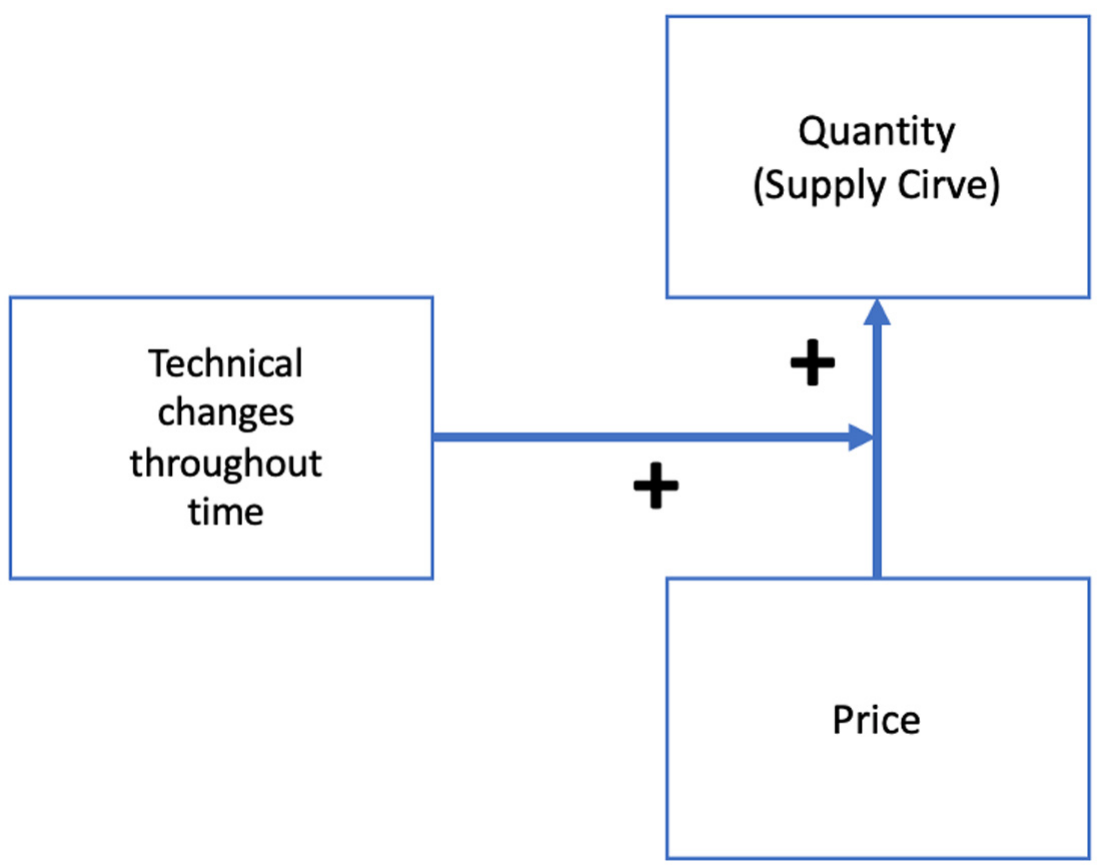
Model of how Tc affect the P(Qs) function.

In the **P(Qs)** function, **Tc** acts as a positive moderator between **Price** and **Production** (quantity), shifting the supply curve to the right ( Figure 7), by increasing production but maintaining price.

**Figure 8.**
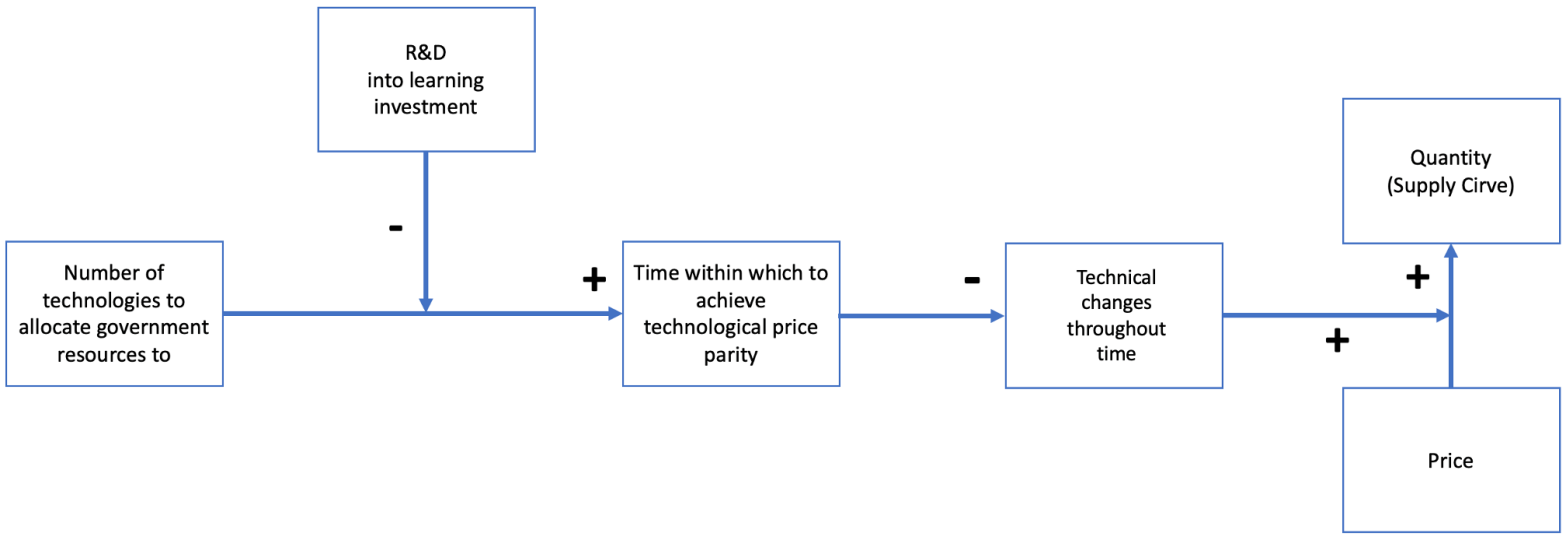
The SURESC Model.

If the timeframe of **Tc** is set to be the timeframe within which we intend to achieve price parity with a competitive technology, we can finally find a relationship between **n** and **Price.** Keeping constant the number of technical changes required to achieve price parity, by diminishing time, we increase **Tc** (and vice-versa). Thus, time within which to achieve technological price parity is negatively related to **Tc**. Having this last relation set, we can propose the final **SURESC** model seen in Figure 8.

**Figure 9.**
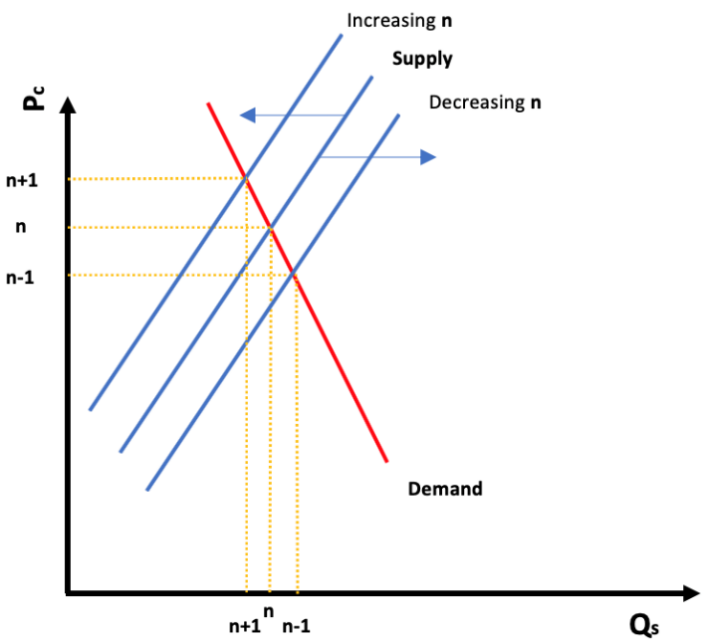
Ideal demand and supply renewable energy curves and shifting effect of selecting fewer technologies.

Figure 9 represents the shift in the supply curve when **n** varies. By increasing **n**, the supply curve will shift to the right, and vice versa, by diminishing **n**, the supply curve will shift to the left (in energy markets, both demand and supply are believed to be inelastic (b <1) ^24, 41, 42^).

## Conclusion

It’s important to underline the fact that there is no perfect solution to the energy transition, that any technology used will have its pros and cons, breakthroughs may come from excluded ones, and that economic frameworks strongly suggest a diversified portfolio of technologies to increase success rates. Nevertheless, among maturing technologies, the application of the **SURESC** **model** could speed-up the energy transition with available technologies. Time is of essence in the battle to save our environment, and we need to adapt rapidly with the tools we have today. Technological breakthroughs can arrive at any point in time, but Earth’s no-return temperature point (estimated within an average increase in global temperatures between 1.5°C to 2°C ^43^) has a set date and is rapidly approaching. Consequently, it’s my understanding that public resources should be used to speed-up the energy transition process by canalizing most of the resources towards an existing maturing technology and leave the private sector to apply resources to find alternative technology solutions while a strong incentivized energy transition is taking place.

Applying the SURESC model would have a profound impact on the entire energy supply chain. First, renewable systems would have to switch from a decentralized model to a centralized one, with the introduction of concepts such as of smart grids and system flexibility (daily and seasonal). The entire chain will have to be standardized (with clear engagement policies) and monitored by a regulatory authority which would guarantee a correct application of the resources and operations. Renewable sources and energy curriers would have to be selected according to geographical regions, efficiency, and power demands among maturing technologies. Alternative technologies would have to be left for the private sector to develop. This is an issue some countries are facing today, and the application of a SURESC model at the government level could help to provide a clear and final long-term energy strategy.

For example, in 1992 Italy announced the first incentive mechanisms to develop renewable sources power systems in the country ^44^. The incentives did not limit the technology used, nor the size, and obliged the energy operators to receive any power produced by any renewable source (single and multiple source). Today, ss a result of this unstandardized energy program, Italy’s major energy transmission grid operators must deal with thousands of small producers from different sources (mostly solar PV and onshore wind), which provide a portion of the electricity to the energy grid in “droplets”, at available production times (which differ from demand times) and where the energy systems itself are not prepared to have neither a flexibility trait nor a smart-grid implementation. The results are:

- Loss of efficiency
- A non-optimized system
- The need for large investments in electrical accumulators
- A disordered availability of power
- A disordered localization of production sites

These constrains will keep renewable energy price high, extending price parity time with brown energy sources. Italy’s energy carrier companies are now aiming to build a centralized energy model which will include a system flexibility trait and a smart management of the receiving power, optimizing the entire system. At the same time, these institutions are requiring to policy makers to develop laws that will regulate production and standardize power influx. This is a partial SURESC model application example, which provides a sense of the problem and where limiting the number of technologies to use would close the model gap. Italy is one of the signatories of the Paris Treaty, and consequently must quadruple its renewable source production by 2030 and be a carbon-free emission country by 2050. A SURESC model implementation could help to achieve these goals faster.

## Recommendations

The SURESC model per se will speed-up the energy transformation process but it may not be enough, as it requires governments coordination with coordinated legislations ^31^ creating non-convex economies with convenience equilibria through R&D and taxes/subsidies. Even though standard economic framework suggests that markets and not policymakers and energy geeks should decide winners and losers, to speed-up the energy shift process, governments should incentive a restrictive technological resources allocation policy to achieve a roadmap designed to implement the SURESC model. Furthermore, investments in new oil and gas explorations and the development of new certified reservoirs should be heavily discouraged. In 2019, new investments made on the oil and gas upstream only, summed up at an astonishing 505B$, compared to 166B$ of subsidies globally allocated to the renewable energy upstream industry, and keeps rising at an average of 4% on a year base ^28^. If that money would have been invested on a clear road map, SURESC could play an important role, and the energy shift could become reality at a faster pace. The issue is not to only mitigate new emissions, but to transition the existing ones to renewable sources and contemporarily quench new energy needs, as we are already producing more CO _2_ than the earth can possibly absorb. Perhaps, a possibility would be to pair the SURESC model to the ineffective carbon credits ^45^ formalized by the Kyoto protocols of 1997, and relaunch them as a new green strategy. This opens the opportunity to new research topics.

## Limitations and future studies

As a preliminary study, this theory-building research is intended to provide a theoretical framework on variables that can potentially affect time to achieve a green energy transition. There is the need to test this model with real data, opening an opportunity for future studies. Furthermore, the study doesn’t account for technological breakthroughs that can be achieved at any point on time and could potentially disrupt the SURESC model itself. Unfortunately, technological breakthroughs are an unpredictable solution for a predicted problem (climate change), while the SURESC model is a predictable solution for a predicted problem. Last, the study does not account for possible economic and political implications on applying a SURESC model at a government level, opening another opportunity for further studies.

## Data availability

All data underlying the results are available as part of the article and no additional source data are required.
