# Supplementary material for: Standardizing a unique renewable energy supply chain: the SURESC Model
Source: F1000Res. 2021 Nov 15;9:1391. Originally published 2020 Dec 3. [Version 3] doi: 10.12688/f1000research.27345.3 (PMC10804050; doi:10.12688/f1000research.27345.3)
Supplement: Peer review history

# Reviewer report: Reviewer response for version 3

**DOI:** 10.5256/f1000research.78536.r229216

**Published:** 2024-01-22

**Authors:** Md. Mustafa Kamal

**Affiliations:** 1 Aligarh Muslim University, Aligarh, Uttar Pradesh, India

The authors proposed the *standardization of a unique renewable energy supply chain* model, defined as the SURESC model, relating the indirect effect of limiting the number of maturing technologies to allocate resources to achieve renewable price parity with conventional energy sources faster. The authors have revised the manuscript and added to what was previously suggested by the reviewers. Now, it can be accepted.

Is the work clearly and accurately presented and does it cite the current literature?

Yes

If applicable, is the statistical analysis and its interpretation appropriate?

Yes

Are all the source data underlying the results available to ensure full reproducibility?

Yes

Is the study design appropriate and is the work technically sound?

Yes

Are the conclusions drawn adequately supported by the results?

Yes

Are sufficient details of methods and analysis provided to allow replication by others?

Yes

Reviewer Expertise:

rnewable energy planning, optimization, microgrid plaaning, renewable intergration

I confirm that I have read this submission and believe that I have an appropriate level of expertise to confirm that it is of an acceptable scientific standard.

# Reviewer report: Reviewer response for version 3

**DOI:** 10.5256/f1000research.78536.r100201

**Published:** 2021-11-15

**Authors:** Jatin Nathwani

**Affiliations:** 1 Waterloo Institute for Sustainable Energy (WISE), University of Waterloo, Waterloo, Ontario, Canada

I have reviewed the revised paper & it is a substantial improvement. I have no further concerns.

Is the work clearly and accurately presented and does it cite the current literature?

Partly

If applicable, is the statistical analysis and its interpretation appropriate?

Partly

Are all the source data underlying the results available to ensure full reproducibility?

Partly

Is the study design appropriate and is the work technically sound?

No

Are the conclusions drawn adequately supported by the results?

No

Are sufficient details of methods and analysis provided to allow replication by others?

No

Reviewer Expertise:

NA

I confirm that I have read this submission and believe that I have an appropriate level of expertise to confirm that it is of an acceptable scientific standard.

# Response

**Published:** 2021-11-15

**Authors:** Emiliano Finocchi

**Affiliations:** LUISS Guido Carli, Italy

Thank you for your time and consideration. Much appreciated!

# Reviewer report: Reviewer response for version 2

**DOI:** 10.5256/f1000research.54508.r92086

**Published:** 2021-09-09

**Authors:** Jatin Nathwani

**Affiliations:** 1 Waterloo Institute for Sustainable Energy (WISE), University of Waterloo, Waterloo, Ontario, Canada

1. This article is not of indexing quality. It conflates many issues and there is little clarity in the flow or, neither is there a logical argument from a key proposition to a convincing conclusion.

 2. There is a wide literature and many citations and references which lack focus.

 3. Although some of the sub-paragraphs in a section, as stand-alone items are correct, as a reader it is difficult to ascertain the linkages or relevance.

 4. The statements on geopolitics of energy are very much reflective of an opinion piece in a newspaper editorial. It is not clear whether there is a cogent flow to the next section and so on.

 5. The section on learning curves is well-known and the idea that R&D will improve learning curves together is well recognized. What is not clear is how the scale of implementation of the technologies will reduce the costs and become competitive with the barriers that the fossil fuels impose on the system. How does any of this link to the SURESC model and the figures are conceptual line diagrams? There appears to be no data behind the figures.

 6. In several sections of the article, there are many citations. These are more or less correct as cited but the question I have is: so what? We know the properties of Hydrogen, we know the properties of ammonia & so on. What is entirely unclear is a case for why or how it may offer a sustainable pathway for a cleaner energy future. 

 7. In summary, this paper is not well written and is not of a high enough standard for indexing. I was expecting a critical analysis of the supply chain of a renewable energy pathway and instead what we have is more a disconnected literature.

Is the work clearly and accurately presented and does it cite the current literature?

Partly

If applicable, is the statistical analysis and its interpretation appropriate?

Partly

Are all the source data underlying the results available to ensure full reproducibility?

Partly

Is the study design appropriate and is the work technically sound?

No

Are the conclusions drawn adequately supported by the results?

No

Are sufficient details of methods and analysis provided to allow replication by others?

No

Reviewer Expertise:

NA

I confirm that I have read this submission and believe that I have an appropriate level of expertise to state that I do not consider it to be of an acceptable scientific standard, for reasons outlined above.

# Response

**Published:** 2021-09-27

**Authors:** Emiliano Finocchi

**Affiliations:** LUISS Guido Carli, Italy

Thank you Jatin, 

 Your comments are welcome. I believe you're right, going through the paper after a while I may understand your perspective. I will revise it and upload a third version.

# Reviewer report: Reviewer response for version 2

**DOI:** 10.5256/f1000research.54508.r79638

**Published:** 2021-03-03

**Authors:** Michel Noussan, Giacomo Falchetta

**Affiliations:** 1 Fondazione Eni Enrico Mattei, Milan, Italy

The author claims that the peer review is “written from a biased source and questions the integrity of the reviewer itself”. We wonder what the reviewer exactly means by “biased source”. We can safely reassure the reviewer that no gender/nationality/affiliation/journal/topic bias affected our screening of the manuscript and that our common aim is that of a constructive process to generate quality scientific content. Our comments might sometimes sound a bit direct as they go ‘right to the point’, but there is no point in taking comments ‘personally’.

 While the author has responded to some of our comments, the changes to the manuscript were limited to minor corrections to some typos. Thus, we still believe that the quality of this paper is not suitable for an international publication and we cannot recommend its indexing. The author presents a “model” but without any description of the calculation process, and its conclusions do not seem to be reproducible. He claims that “the theoretical model I present should be tested with empirical data on a second study (including a sensitivity analyses), opening an opportunity rather than a flaw.”. We appreciate this indication and we believe this should be reflected clearly both in the introduction (aims and scope of the paper) and more widely in the conclusion and discussion of future research.

 Moreover, the author argues that some figures are drawn directly from Shayegh et al. Generally it is correct to build on assumptions from others’ peer reviewed work BUT 1) these assumptions should not be taken ‘blindly’ but critically evaluated in the context of the work carried out and 2) looking at Shayegh et al.’s figure 2 (from which the author builds her own figure 2), I notice they do indeed have units on the axis, which are still missing in this paper’s figure 2.

 One key aspect that we highlighted is the efficiency of the ammonia supply chain, which is at the basis of the suitability of this solution, especially when compared with a hydrogen pathway, proposed by the author in his model. Specifically, referring to the 72% efficiency, this value is not a result of the Nayak-Luke work mentioned by the author, since this work is simply referencing this value, which is in turn obtained in the work by “Wang, G., Mitsos, A. and Marquardt, W. (2017), Conceptual design of ammonia‐based energy storage system: System design and time‐invariant performance. AIChE J., 63: 1620-1637. https://doi.org/10.1002/aic.15660 ^1^”. In this work, as specified by the title and the methodology, a simulation model is proposed with a theoretical optimization supporting a conceptual design. In fact, two separate results are given: 72% and 64% (with a set of assumptions given by the authors), and citing only the highest one is already a questionable choice. We are not questioning the validity of this study here. However, we are questioning the choice of the author of basing all his model on a single value, from a theoretical study on a potential pathway (which is not implemented today in real operations), without further research. From a scientific point of view, a proper sensitivity approach would include other studies. As an example, please consider the paper “S. Giddey, S. P. S. Badwal, C. Munnings, and M. Dolan, Ammonia as a Renewable Energy Transportation Media, ACS Sustainable Chemistry & Engineering 2017 5 (11), 10231-10239, DOI: 10.1021/acssuschemeng.7b02219 ^2^”, which presents a more comprehensive description of different ammonia pathways, with roundtrip efficiency values from existing and potential applications in the range 19%-50% (when considering best-case scenarios for each application).

 Concerning the discussion on the comparative advantage, the author claims: “I assume that in a global warming scenario, we all win, or we all lose” as a justification for the paper not considering political implications and assuming the collaboration of nations rather than competition among them. Unfortunately, this is not an assumption which seems to describe the current status of climate negotiations, where incentives of countries are not aligned because of heterogeneous social cost of carbon (Ricke et al., https://www.nature.com/articles/s41558-018-0282-y ^3^) and impacts (Pretis et al. https://royalsocietypublishing.org/doi/10.1098/rsta.2016.0460 ^4^) across countries. While we appreciate that this aspect is hard to model explicitly, we believe that it should be highlighted as a limitation of the proposed model.

Is the work clearly and accurately presented and does it cite the current literature?

Partly

If applicable, is the statistical analysis and its interpretation appropriate?

Partly

Are all the source data underlying the results available to ensure full reproducibility?

No

Is the study design appropriate and is the work technically sound?

No

Are the conclusions drawn adequately supported by the results?

No

Are sufficient details of methods and analysis provided to allow replication by others?

No

Reviewer Expertise:

Michel Noussan: energy technologies, energy policy; Giacomo Falchetta: energy economics, energy modelling

We confirm that we have read this submission and believe that we have an appropriate level of expertise to state that we do not consider it to be of an acceptable scientific standard, for reasons outlined above.

## References

[1] : Conceptual design of ammonia-based energy storage system: System design and time-invariant performance. *AIChE Journal* .2017;63(5) : 10.1002/aic.15660 1620-1637 10.1002/aic.15660

[2] : Ammonia as a Renewable Energy Transportation Media. *ACS Sustainable Chemistry & Engineering* .2017;5(11) : 10.1021/acssuschemeng.7b02219 10231-10239 10.1021/acssuschemeng.7b02219

[3] : Country-level social cost of carbon. *Nature Climate Change* .2018;8(10) : 10.1038/s41558-018-0282-y 895-900 10.1038/s41558-018-0282-y

[4] : Uncertain impacts on economic growth when stabilizing global temperatures at 1.5°C or 2°C warming. *Philos Trans A Math Phys Eng Sci* .2018;376(2119) : 10.1098/rsta.2016.0460 10.1098/rsta.2016.0460 29610370 PMC5897830

# Reviewer report: Reviewer response for version 1

**DOI:** 10.5256/f1000research.30219.r76545

**Published:** 2021-01-18

**Authors:** Michel Noussan, Giacomo Falchetta

**Affiliations:** 1 Fondazione Eni Enrico Mattei, Milan, Italy

The aim of the paper may be of interest, given the importance of the subject in the current debate on the energy transition. Unfortunately, the quality of this paper is not suitable for an international publication. The author presents a theoretical “model” (I would rather call it framework, by the way) but without any description of the calculation process (which we suspect was not carried out, i.e. the framework has not been tested against real world data), and its conclusions do not seem to be reproducible. The selection of the ‘most suitable technology’ at page 18 seems not to be grounded by a scientific analysis but just based on a qualitative discussion, which is at odds with introducing what the author calls ‘a model’. Also, there is poor documentation on some key parameters/values considered. In particular, most of the figures for ammonia do not seem to be reliable. The efficiency of 72% is wrong, and the cited source (50) is not dealing with ammonia at all. Considering the ammonia supply chain, a total efficiency of 72% seems rather hard to reach (especially compared to the lower figures for hydrogen proposed by the author), as hydrogen must still be produced via electrolysis, and at the end electricity needs to be generated from ammonia to be supplied to the users, just like for the hydrogen supply chain. In addition, the energy consumption of the process needs to be considered.

 In general, any proposed model needs to be reproducible, clearly describe the input data and the calculation procedures, to support a sound presentation of the results. Moreover, any result needs to include a description of the possible errors and approximations, by presenting a sensitivity analysis that highlights the possible variations. This is particularly true for complex energy systems, whose parameters show significant variations over time and in different world regions. All these aspects are missing in this manuscript, and thus I think that this research paper is not suitable for publication in its present form.

 Another general comment to the framework: how about comparative advantage in generation and production + trade between countries? The introduced framework may be okay in a one-nation world with even distribution of RE potential. But if country A has significant comparative advantage for producing PV modules and generating electricity with PV and country B has a similar comparative advantage for wind turbines, then cumulative investment requirements are lower if country A targets investments in PV and country B targets investments in wind and they trade the produced electricity, rather than if both invest massively in PV (this is simply Ricardo’s comparative advantage).

 There are other issues in the manuscript.

 Although it is nice to see a paper written in an ‘entertaining’ language, sometimes the tone of the papers does not look suitable for a scientific publication. Moreover, there are several instances where important references are not cited:

- Section ‘Geopolitics of the black gold’ à at least cite an IPCC report when illustrating the link between energy consumption and climate change.
- Discussion on ‘The Dutch Disease’ à cite and critically discuss academic literature on the resource curse please

Page 4: “I propose that there is a direct positive relation…” à this is the core assumption of the paper and it should be better defended with some evidence, e.g. from the literature! I understand you later introduce a conceptual framework to back this assumption up, but the model is not validated or tested on real world data, so you could sketch a framework for any arbitrary assumption you wish to make. At least say ‘I hypothetise that’.

 Page 4: “non-convex economies” : define what they are perhaps citing some literature, it is not obvious

 Section ‘And green we go…’, line ‘(with exception of hydropower)’ à and biomass; also e.g. CSP necessitates significant amounts of water.

 Some charts are provided without numbers in axes (e.g. Figure 2), and the sources are not always properly referred to. In particular in Figure 2 the author should either specify it is a sketch or put values of the axes. Also, even if it is a sketch, put a source. In current form, it is not acceptable from a scientific perspective. Also, I question the linearity of these "curves"! At least specify immediately that it is a sketched graph that neglects the declining returns (second derivative negative) to learning-by-doing.

  Moreover, a fixed cost for fossil generation of 50 €/MWh appears rather approximated: there is in fact a range of costs depending on multiple variables, including a variation across fossil sources, including natural gas and coal (the latter being usually cheaper).

 Also, below Figure 2: “Solar PV has a learning quotient of 23%, intercepting fossil fuel line (price parity) with a future cumulative quantity (power deployed) at over 1000 TWh” à future when and where?!

 In some cases, limited examples are given that are not representing the complexity of the situation. In the section “Dangers of unbalancing the energy geopolitics”. There are also countries that are actually decreasing their carbon footprint. The example of the US is important, but additional figures from other countries may help to have a broader perspective. While it depends on producer vs. consumer perspective, it is still a false statement for the EU (see https://ec.europa.eu/eurostat/statistics-explained/images/3/35/CO2_emissions_%E2%80%94_production_and_consumption_perspective%2C_EU-28%2C_2008-2015_%28index_2008_%3D_100%29.png)

 Section “Energy Streams”: this section reports a limited perspective. It is true that energy cannot be created or destroyed, as stated by the first principle of thermodynamics. However, the second principle states also that the quality of energy always decreases, due to the increase of entropy, as in real world energy conversions are non-reversible. Without considering those aspects, this discussion is misleading.

 Section “Downstream”: the author states “We shall therefore not account for the transportation sector in this research as it will shape and adapt by itself according to the external environment and clients’ needs”. This sentence is very hard to justify, since the EV market is currently heavily pushed by environmental policies and public incentives. The author also states that transport “will be the strongest growing energy demand sector in the future”. Thus, overlooking it seems not a satisfactory choice. The authors should also discuss the possible limitations on shifting towards electricity for specific applications, such as buildings heating. While heat pumps are promising, they are not directly applicable in all contexts, and they may require a significant improvement of power networks’ installed capacity, especially in some specific time frames.

 There are other minor typos/errors. “concentrated solar PV” is a typo, as concentrated solar power is a different technology than photovoltaic (PV). Cumulative power deployed (describing Figure 2) cannot be expressed in TWh, which is a unit for energy and not for power.

 Other typos/language issues:

- Page 3: “Not least, the impact to the environment for such amounts of fossil-fuel consumptions is well documented8–11 and accounts” à “Not least, the impact on the environment for such amounts of fossil-fuel consumption is well documented8–11 and is responsible for…”
- Page 4: “The randomization nature of the world’s natural resources distribution” à randomization is improper; I would say uneven distribution
- Page 4: “lacking 11 TWh of power generation that has been supplied by carbon fuels” à typo, should be 101 TWh
- Page 4: “The literature seam to point” à seems to point

Is the work clearly and accurately presented and does it cite the current literature?

Partly

If applicable, is the statistical analysis and its interpretation appropriate?

Partly

Are all the source data underlying the results available to ensure full reproducibility?

No

Is the study design appropriate and is the work technically sound?

No

Are the conclusions drawn adequately supported by the results?

No

Are sufficient details of methods and analysis provided to allow replication by others?

No

Reviewer Expertise:

Michel Noussan: energy technologies, energy policy; Giacomo Falchetta: energy economics, energy modelling

We confirm that we have read this submission and believe that we have an appropriate level of expertise to state that we do not consider it to be of an acceptable scientific standard, for reasons outlined above.

# Response

**Published:** 2021-01-20

**Authors:** Emiliano Finocchi

**Affiliations:** LUISS Guido Carli, Italy

Denigrating other people work is not a constructive way of doing a peer review. Contents could be discussed without the need for an offensive use of the language. This review seams to be written from a biased source and questions the integrity of the reviewer itself. When the reviewer refers to my paper as written in a “entertaining language”, I would like to underline that this paper is intended not only for the academics, but for practitioners too. Many definitions are written on purpose to help the non-academic reader.

 Down to content:

 First, this is a **Theory-Building Research** (for more information on theory-building please consult: *Lynham, Susan. (2002). The General Method of Theory-Building Research in Applied Disciplines. Advances in Developing Human Resources*), and not an empirical study, where theories are built on other researchers’ work following a rigorous scientific reasoning. The theoretical model (not a framework) I present should be tested with empirical data on a second study (including a sensitivity analyses), opening an opportunity rather than a flaw. Real data can support or not support the model, in either case, it can’t question methodology.

 To build the model, I have started from Shayegh et al work ( *Shayegh S, Sanchez DL, Caldeira K: Evaluating relative benefits of different types of R&D for clean energy technologies*) and mathematically calculated my way through. Calculations can be found from page 5 to page 8. The figures (not sketches) are generic, based on previous studies, and having a scale attached to the axes would not change the result. Figure 2 in particular, is drawn from Shayegh et al work. Doubting this figure, means to doubt a published work and should not be mentioned in this review. Moreover, it’s well written that the starting point which Figure 2 was drawn, is driven by Shayegh et al work. In my second version I will make this clearer. In fact, when the reviewer states on Figure 2 that “I question the linearity of these "curves” “he is questioning the linearity of Shayegh et al work, not mine. It is clear that the reviewer did not spent time to read Shayegh et al work at all.

 When the reviewer comments “Figure 2: “Solar PV has a learning quotient of 23%, intercepting fossil fuel line (price parity) with a future cumulative quantity (power deployed) at over 1000 TWh” à future when and where?!” misses the entire point of what I am trying to support. When and where are not the questions I am answering. “How fast or how slow compared to”, would be the correct question. The reviewer is asking questions I myself did not ask in the paper.

 Moreover, the reviewer states that the model is not replicable, but it doesn’t explain why (it doesn’t help much).

 When the reviewer states that “The selection of the ‘most suitable technology’ at page 18 seems not to be grounded by a scientific analysis but just based on a qualitative discussion” I rather question if the reviewer acknowledges that a qualitative analysis is indeed a scientific analysis.

 When the reviewer refers to ammonia as “The efficiency of 72% is wrong” I may understand his doubts, as there is an error on that reference. The correct reference 50 is “ *Nayak-Luke, R., Bañares-Alcántara, R., & Wilkinson, I. (2018). “Green” ammonia: impact of renewable energy intermittency on plant sizing and levelized cost of ammonia. Industrial & Engineering Chemistry Research, 57(43), 14607-14616* *”. *Nayak-Luke et al work states green ammonia is achievable at 72%. This is a published work. If we shall question every published work without another publishable work that dismisses the previous one, why are we so busy to publish? Based on *Nayak-Luke* work, the reviewer is wrong. I will correct all references in my second version.

 When the reviewer states “Section ‘Geopolitics of the black gold’ à at least cite an IPCC report” what it really means? What is “at least” for him? Are my sources not enough? Why? “At least” is not a constructive comment if it’s not backed up.

 The reviewer’s comment on the Dutch Disease is not clear. I really did not understand, as it was a dismissible reference, not part of the main reasoning. He could have simply advised to remove the comment. I don’t believe there is the need to remove it, I will keep it in my next version.

 The reviewer’s comment on non-convex economies is understandable. I will explain more on the subject in my second version of the study.

 In terms of competitivity (Riccardo’s comparative advantage), the study argues that if countries cooperate and basket all resources for a common technology, that technology will achieve competitiveness at a faster pace. Rather than building wind turbines in your country, use the resources to build solar PV in other countries in a cooperative context. This implies building power generation in a country and power usage in others. If the reviewer had focused on the paper, he would understand that the framework within which the model is built, does not consider political implications. I will make it explicit in the second version that the model foresees the collaboration of nations rather than competition among them. The entire aim of the paper is to push for cooperation and collaboration, and not to spike competitiveness. Riccardo’s paper assumes winners and losers, in a competitive environment, which in my paper, I assume that in a global warming scenario, we all win, or we all lose.

 When the reviewer states that “the second principle states also that the quality of energy always decreases, due to the increase of entropy, as in real world energy conversions are non-reversible. Without considering those aspects, this discussion is misleading.” I don’t see where is misleading. Maybe the reviewer could share with us how this is misleading.

 Section “downstream”, as suggested by the reviewer, opens to another (or many), new studies. Nevertheless, the reviewer is correct, and I shall explicitly define the boundaries of the study. I will correct this aspect in the second version.

 “Other type of language errors” section of the review will be amended as suggested in the second version of the paper.

 Thank you
